# Cu_7.62_Bi_6_Se_12_Cl_6_I: Discovery of a Low Band Gap, Low Thermal Conductivity Mixed-Anion Material

**DOI:** 10.1021/acs.chemmater.5c02919

**Published:** 2026-02-24

**Authors:** Cara J. Hawkins, Batoul Almoussawi, Jan P. Scheifers, Manel Sonni, Aeshah A. Almushawwah, Troy D. Manning, Marco Zanella, Craig M. Robertson, Luke M. Daniels, Tim D. Veal, John B. Claridge, Matthew J. Rosseinsky

**Affiliations:** † Department of Chemistry, Materials Innovation Factory, 4591 University of Liverpool, 51 Oxford Street, Liverpool L7 3NY, U.K.; ‡ Leverhulme Research Centre for Functional Materials Design, Materials Innovation Factory, University of Liverpool, 51 Oxford Street, Liverpool L7 3NY, U. K; § Stephenson Institute for Renewable Energy and Department of Physics, 4591 University of Liverpool, Liverpool L69 7ZF, U.K.; ∥ Department of Physics, Faculty of Science and Humanities in Al-Dawadmi, Shaqra University, Shaqra 11911, Saudi Arabia

## Abstract

The exploration of higher-dimensional chemical phase spaces and the synthesis of novel compounds can be achieved by applying a multiple-anion approach to materials discovery. The ability to combine and tune the stoichiometry of anions in a material can enable enhanced control of both the physical and electronic structures, providing a strategy for the modification of the properties of new materials being developed for a variety of applications, including solar absorbers and thermoelectrics. Here, we report the synthesis of Cu_7.62_Bi_6_Se_12_Cl_6_I, a quadruple-anion (Se^2–^, (Se_2_)^2–^, Cl^–^, I^–^) material within the Cu–Bi–Se–Cl–I phase space. Crystal growth reactions yield black, needle-like crystals, which exhibit a highly anisotropic and complex structure containing the four distinct anion types, solved from single-crystal X-ray diffraction data. Compositional analysis confirms the complex material stoichiometry, and a low band gap of 0.94(5) eV is measured to understand the potential for solar-absorbing applications. Cu_7.62_Bi_6_Se_12_Cl_6_I has a low thermal conductivity of 0.25(2) W K^–1^ m^–1^, which is attributed to multiple structural features via analysis of experimental heat capacity data and is achieved through the diversity in bonding that is accessed through the combination of four different types of anion.

## Graphical abstract

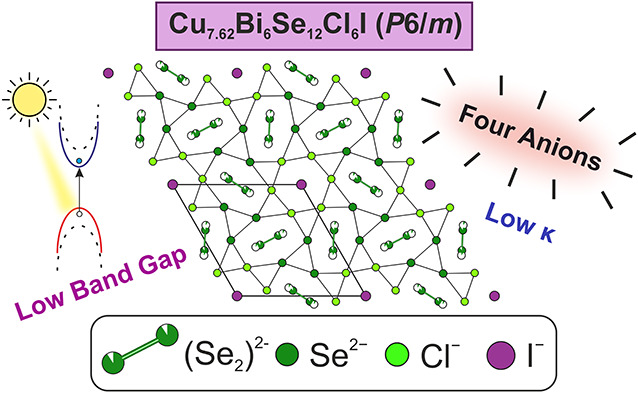

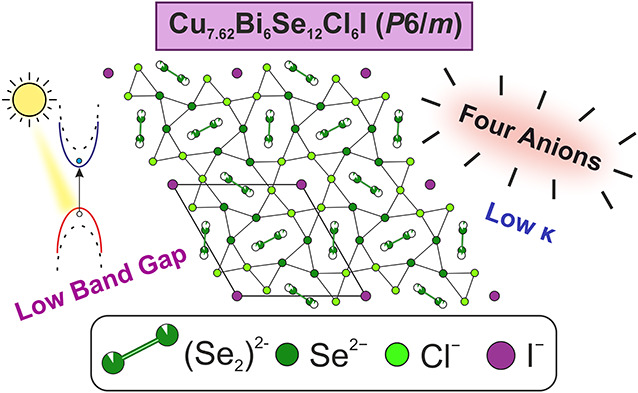

## 1 Introduction

The application of mixed-anion chemistry to materials discovery has become a significant area of research in recent years.^1,2^ The inclusion of multiple anions into a single structure not only enables exploration of larger compositional phase spaces but also facilitates greater tunability of both structure and thus properties.^3^ Despite this, the chemical spaces that utilize multiple anion types are typically much less explored relative to phase spaces based on single anions.

Halide-chalcogenide (or “chalcohalide”) materials containing at least one halogen and one chalcogen have emerged as a promising option for a variety of applications.^4^ In particular, mixed-metal chalcohalide materials containing lone pair cations such as Bi^3+^ and Sb^3+^ have gained attention for solar-absorbing applications,^5,6^ owing to their desirable optoelectronic characteristics,^7^ defect-tolerant electronic structure,^8^ and enhanced environmental stability in comparison with materials like lead halide perovskites (LHPs).^9^ Examples include Sn_2_SbS_2_I_3_ ^10^ and Pb_2_SbS_2_I_3_,^11^ which have achieved Power Conversion Efficiencies (PCEs) of 4.04% and 3.12% in prototype solar cells, respectively. Furthermore, such chemistries have shown promise for thermoelectric applications, with very low thermal conductivities commonplace in these materials, which result from structural anisotropy often induced when incorporating multiple anion types and stereochemical activity of a heavy lone pair cation.^12–14^ In particular, asymmetric local coordination environments, driven by the mixed-anion chemistry, can generate localized oscillators in the structure, which inhibit thermal transport.^15^

More recently, combinations of *d* ^10^ transition metals (e.g., Cu^+^ and Ag^+^) alongside Bi^3+^ in quaternary (Cu, Ag)–Bi–Ch–X spaces (Ch = S^2–^, Se^2–^, Te^2–^; X = F^–^, Cl^–^, Br^–^, I^–^) have been explored for potential optoelectronic applications, yielding materials such as AgBiSCl_2_,^16^ CuBiSCl_2_,^17^ and CuBiSeCl_2_.^18^ Thus far, of all the quaternary compounds in this space studied for potential optoelectronic applications, X = Cl^–^. The inclusion of I^–^ can be highly beneficial to the optoelectronic properties of semiconductor materials by increasing band dispersions and improving charge carrier mobilities.^19^ The inclusion of additional anions in the exploration space (e.g., Cu–Bi–Se–Cl–I) thus presents an opportunity due to the additional flexibility for tuning of chemical structure and material properties, as demonstrated in lead halide perovskites for solar absorption applications,^20^ and thermoelectric materials,^21^ or in the realization of significantly different structures in materials that contain similar cations.^22^

Here, the synthesis of Cu_7.62_Bi_6_Se_12_Cl_6_I, the first quinary material in the Cu–Bi–Se–Cl–I phase space, is reported. The structure is characterized through both single-crystal and powder X-ray diffraction; Cu_7.62_Bi_6_Se_12_Cl_6_I is structurally related to the known material Cu_7.4_Bi_6_Se_12_Cl_7_ but displays significantly reduced Cu^+^ disorder because of modifications to the cationic bonding environments, defined by the introduction of I^–^ into the structure. The structure solution from single-crystal X-ray diffraction (SCXRD), combined with oxidation state analysis using X-ray photoelectron spectroscopy (XPS), reveals the presence of both Se^2–^ and (Se_2_)^2–^ species, making Cu_7.62_Bi_6_Se_12_Cl_6_I a quinary compound containing four distinct anions. The optoelectronic properties are characterized to understand its potential as a solar absorber material, and the band alignments are determined via XPS. Cu_7.62_Bi_6_Se_12_Cl_6_I is found to exhibit a low thermal conductivity at room temperature, arising from a combination of bonding anisotropy and localized oscillators.

## 2 Experimental Section

### 2.1 Reagents

CuCl (99.995%, Merck), CuI (99.998%, Thermofisher Scientific), Bi_2_Se_3_ (>99.995%, Sigma-Aldrich), Cu powder (99.9%, Sigma-Aldrich), and Se powder (>99.5% Sigma-Aldrich) were used without further purification. Reagents were stored and handled in an Ar-filled glovebox with <0.1 ppm of O_2_ and <0.1 ppm of H_2_O.

### 2.2 Synthesis of Cu_7.62_Bi_6_Se_12_Cl_6_I

Following our recent report on CuBiSeCl_2_,^18^ reactions targeting related stoichiometries with the inclusion of different cations were performed, along with the addition of an iodine transport agent to promote crystal growth. This included a CuZnBiSe_2_Cl_2_ composition; for this reaction, a stoichiometric mix of reagents (CuCl (99.995%, Merck), ZnCl_2_ (99.999%, Merck), Bi_2_Se_3_ (>99.995%, Sigma-Aldrich), and BiCl_3_ (99.995%, Sigma-Aldrich)) was ground together in an agate mortar and loaded into a quartz ampule of dimensions 1 cm × 20 cm, along with ∼10 wt % I_2_. The ampule was evacuated to 1 × 10^–3^ mbar and sealed. The ampule was placed horizontally into a high-temperature oven before heating to 430 °C using a ramp rate of 5 °C/min, dwelling at this temperature for 10 h, and cooling to 200 °C using a ramp rate of 0.1 °C/min, before shutting off the oven and allowing it to cool to room temperature. The sample obtained after firing was a black melt with black, needle-like single crystals. These crystals were used for structure determination by SCXRD at beamline I-19, and the crystal structure was solved and found to exist as the previously unreported material Cu_7.62_Bi_6_Se_12_Cl_6_I. Energy Dispersive X-ray Spectroscopy (EDX) analysis measured on single crystals of Cu_7.62_Bi_6_Se_12_Cl_6_I confirmed the absence of any Zn in the structure (Figure S0).

To confirm these initial results, further experiments were carried out targeting the nominal Cu_7.62_Bi_6_Se_12_Cl_6_I stoichiometry without any Zn-containing reagents. Samples of Cu_7.62_Bi_6_Se_12_Cl_6_I were synthesized on a 1 g scale by grinding stoichiometric amounts of the reagents listed in Section 2.1 in an agate mortar before loading the mixture into a flame-dried quartz ampule of dimensions 1 cm × 20 cm. The ampule was evacuated to 1 × 10^–3^ mbar and sealed. The ampule was then placed horizontally into a high-temperature oven before heating using the same temperature profile as the exploratory reactions. The sample obtained after firing was a black melt with black, needle-like single crystals that were used for structural determination by SCXRD or ground into a powder for other analysis methods. The results from SCXRD, powder XRD, and EDX/WDX analysis revealed that the Zn-free reactions consistently produced Cu_7.62_Bi_6_Se_12_Cl_6_I single crystal and bulk powder samples with the same structure and composition as the initial Zn-containing reactions (S1–S3, S13–14, S17).

### 2.3 Single-Crystal X-ray Diffraction (SCXRD)

A black single crystal of Cu_7.62_Bi_6_Se_12_Cl_6_I with a needle-like shape was selected under a polarizing microscope and then studied by SCXRD on beamline I19, Diamond Light Source, U.K. using silicon double crystal monochromatic synchrotron radiation (λ = 0.6889 Å, Pilatus 2 M detector).^23^ Synchrotron data were collected with the sample at 100 K. Cell refinement and data reduction were performed using Xia^24^ and Dials programs.^25^ Using Olex2,^26^ the structure was solved with the SHELXT^27^ structure solution program using Intrinsic Phasing and refined with the SHELXL^28^ refinement package using Least Squares minimization. The occupancies of the Bi1/Bi2 and Se1/Se2 sites were each constrained to sum to 1, respectively. The Se1/Se2 sites are constrained such that they have the same occupancy as the corresponding Bi sites. This is justified by the fact that the Bi1–Se2 and Bi2–Se1 cases of coordination are not possible because the resulting Bi–Se bonding distances are outside the range of reasonable bond lengths (Bi1–Se2: 3.365(5) Å; Bi2–Se1: 3.493(10) Å), as supported by analysis of Bi–Se bond-length histograms reported in the Inorganic Crystal Structure Database (ICSD), which indicate that the majority of compounds exhibit Bi–Se bond lengths (l) of 2.7 < *l* < 3.2 Å. In addition to this, independent refinement of the occupancies without these constraints in place yields values within error to those reported here: 0.906(1)/0.094(1) for Bi1/Bi2 and 0.900(5)/0.100(5) for Se1/Se2. The final residual factors converged to *R*_1_ = 0.0128 and wR_2_ = 0.0290 for reflections with I > 2σ (I). The final refinement parameters, isotropic and anisotropic thermal displacement parameters, and main bond distances from the single crystal solution are listed in the Supporting Information S1–S3.

The computer program BayMEM^29^ was used to run maximum entropy method (MEM) calculations and produce electron density maps for Cu_7.62_Bi_6_Se_12_Cl_6_I on a grid of 300 × 300 × 300 voxels. An input file of SCXRD diffraction data from a crystal of Cu_7.62_Bi_6_Se_12_Cl_6_I contained the observed structure factors. The structural refinement model developed in Olex2 provided the calculated structure factors. Electron densities were visualized using VESTA.^30^

### 2.4 Powder X-ray Diffraction (PXRD)

Preliminary phase identification of powder samples and pressed pellets was carried out using an X’Pert Panalytical diffractometer with monochromatic Co Kα_1_ radiation (λ = 1.788965 Å) in Bragg–Brentano geometry. 16 h scans were conducted on a Bruker D8 Advance with monochromatic Cu Kα_1_ radiation (λ = 1.54056 Å) in Debye–Scherrer geometry to obtain high-quality data for Rietveld analysis of the bulk powder, with the sample sealed inside 0.1 mm diameter borosilicate capillaries in an Ar-filled glovebox. Rietveld refinements against Cu_7.62_Bi_6_Se_12_Cl_6_I PXRD data were conducted using TOPAS Academic V5.24.^31^ The background, lattice parameters, atomic positions, and atomic displacement parameters were refined against the data; a Chebyshev polynomial function was used to model the background, and the peak shapes were modeled using Stephens hexagonal functions.^32^ Structural refinement parameters are available in the Supporting Information (S14)

### 2.5 Raman Spectroscopy

Raman data were collected using a Raman microscope (Renishaw, inVia Reflex coupled with an inverted Leica microscope), with a 785 nm laser as the excitation source (power <300 μW), focused onto the sample using a ×50 objective (Olympus). The collected Raman spectra were baseline-corrected. The spectral measurement time was 1 s with 10 accumulations to ensure a good signal-to-noise ratio and well-resolved peaks.

### 2.6 Compositional Analysis

Scanning Electron Microscopy (SEM) was performed on a Tescan S8000 instrument equipped with a Quorum PP3010 Cryo-FIB/SEM Preparation System. Powder and pellet samples were deposited over an adhesive carbon tape stuck on an aluminum SEM stub, and samples were coated with a thin layer of platinum to reduce charging effects. The sample preparation, handling, and transfer were performed in air-free gloveboxes and vacuum transfer holders. Energy Dispersive X-ray Spectroscopy (EDX) and Wavelength Dispersive X-ray Spectroscopy (WDX) were performed on the same instrument using X-Max^N^ and Wave detectors from Oxford Instruments. SEM-EDX was used to confirm the composition of Cu, Bi, Se, and I, and SEM-WDX was used to quantify the relative Bi/Cl ratio to account for the overlapping Bi^3+^ and Cl^–^ peaks in the EDX spectra. EDX correction factors and WDX calibration for the different elements were estimated by measuring the EDX and WDX spectra of appropriate standard materials. Standard purity was confirmed using X-ray diffraction and electron microscopy. Quantification was performed using Aztec software.

### 2.7 Transmission Electron Microscopy (TEM)

Transmission Electron Microscopy (TEM) images were obtained using a JEOL2100+ microscope operating at 200 kV, equipped with an SDD detector from Oxford Instruments (Model: X-Max 65T, featuring a 65 mm^2^ detection surface). The samples were dispersed on carbon-coated gold TEM grids and inserted by using a single-tilt holder designed for air-sensitive materials.

### 2.8 Spark Plasma Sintering

Dense pellets (∼93% theoretical density) were obtained by spark plasma sintering (SPS) of phase-pure Cu_7.62_Bi_6_Se_12_Cl_6_I powder at 300 MPa and 230 °C for 5 min in a 10^–3^ mbar vacuum using a commercial Thermal Technology LLC DCS10 furnace. Powder samples (∼0.70 g) were pressed in a 10 mm diameter graphite-foil-lined tungsten carbide die set (with 6% Co binder). Heating and pressure ramp rates were set to 20 °C/min and 100 MPa/min, respectively. The temperature was monitored through a borehole on the side of the die set by using a thermocouple. After being pressed, the pellets were lightly polished with SiC paper to remove the graphite foil from the pellet surface.

### 2.9 UV–Vis Spectroscopy

Diffuse reflectance of Cu_7.62_Bi_6_Se_12_Cl_6_I powder was measured by using an Agilent Cary 5000 instrument between 200 and 2500 nm with a step size of 1 nm. Calibration to 100% and 0% reflectance was performed prior to measurement using a PTFE standard and a light trap, respectively. The band gap was determined from a Tauc plot using the method described by Makuła et al.^33^

### 2.10 X-ray Photoelectron Spectroscopy (XPS)

XPS data were collected on Cu_7.62_Bi_6_Se_12_Cl_6_I powder using inert atmosphere transfer in a Thermo NEXSA XPS fitted with a monochromatic Al Kα X-ray source (1486.6 eV), a spherical sector analyzer, three multichannel resistive plates, and 128-channel delay line detectors. All data were recorded at 72 W with an X-ray beam size of 400 × 200 μm. Survey scans were recorded at a pass energy of 200 eV, and high-resolution scans were recorded at a pass energy of 40 eV. Electronic charge neutralization was achieved using a dual-beam low-energy electron/ion source (Thermo Scientific FG-03). The ion gun current was 150 μA, and the ion gun voltage was 45 V. All sample data were recorded at a pressure below 10^–8^ Torr and at 21 °C. Data were analyzed using CasaXPS v2.3.24PR1.0.^34^ Peaks were fit with a Shirley background prior to component analysis using Gaussian–Lorentzian (GL) peaks in a ratio of 50:50. All binding energies were measured with respect to the Fermi edge of a Ni foil reference sample.

Determination of the band alignments of Cu_7.62_Bi_6_Se_12_Cl_6_I was conducted in an ultrahigh vacuum (UHV) chamber operating at a base pressure of 1 × 10^–9^ mbar. Core levels and the valence band were probed by using a SPECS monochromatic Al Kα X-ray source (1486.6 eV). The experiments were performed at an X-ray power of 180 W, using a PSP Vacuum Technology electron-energy analyzer operating at a pass energy of 10 eV. The secondary electron cutoff (SEC) measurements used a lower operating power of 15 W and a pass energy of 2 eV. A bias of −10 V was applied to the sample to separate the spectrometer response.

### 2.11 Properties Measurement

SPS-prepared pellets were cut into semicircles with ∼1.03 mm thickness and ∼4.90 mm radius by using a low-speed diamond-blade saw for thermal conductivity measurements. Copper electrodes were attached to the pellets using Ag epoxy and left to dry overnight. The offcuts from the pellets were used for powder diffraction and compositional analysis. The thermal conductivity was measured between 2 and 293 K using two-probe geometry on a pellet of Cu_7.62_Bi_6_Se_12_Cl_6_I using the Thermal Transport Option (TTO) on a Quantum Design Physical Property Measurement System (PPMS).

Heat capacity measurements were performed on a small fragment of a dense pellet, with a mass of 0.0167 g, of Cu_7.62_Bi_6_Se_12_Cl_6_I using the Heat Capacity Option (HCO) on the PPMS. The sample was mounted using N grease. An addenda measurement was performed on the sample holder and grease prior to mounting and measuring the pellet fragment. Both the addenda and pellet fragment measurements were performed between 2 and 300 K.

### 2.12 Air Stability Measurements

The air stability of Cu_7.62_Bi_6_Se_12_Cl_6_I was determined by evenly distributing the powder onto a glass slide lightly covered in grease and measuring PXRD data at specific time intervals.

## 3 Results and Discussion

### 3.1 Structural Description

The structure of Cu_7.62_Bi_6_Se_12_Cl_6_I adopts hexagonal *P*6/*m* (175) symmetry with lattice parameters *a = b =* 15.0289(1) Å, *c =* 4.0145(1) Å. Figure 1 depicts an overview of the structure of Cu_7.62_Bi_6_Se_12_Cl_6_I, solved from single-crystal data measured at 100 K. The structure of Cu_7.62_Bi_6_Se_12_Cl_6_I consists of a rigid framework of corner-sharing CuSe_4_ tetrahedra, connected in the *a-b* plane through a common Se3 vertex and/or diselenide bridges (Figure 1(b)), and in the *c* direction via a shared Se2 vertex. These CuSe_4_ tetrahedra share edges with neighboring monocapped trigonal prism BiSe_3_Cl_4_ environments, which themselves share edges to form “tunnels” along the *c* axis (Figure 1(c)). The channels generated by these BiSe_3_Cl_4_ tunnels are filled by I^–^ positions and disordered Cu^+^ positions, which form face-sharing CuCl_2_I_2_ tetrahedra (Figure 1(d)). Each neighboring tunnel is linked by bridging diselenide bonds. Quinary Cu_7.62_Bi_6_Se_12_Cl_6_I is isostructural with the reported quaternary compound Cu_7.4_Bi_6_Se_12_Cl_7_.^35^ The formula Cu_7.62_Bi_6_Se_12_Cl_6_I can be approximated as (BiSeCl)_6_·(CuSe)_6‑x_(Cu_2_Se)_*x*_·CuI to reflect the key structural themes of this compound and relate them to known binary and ternary phases.

**Figure 1:**
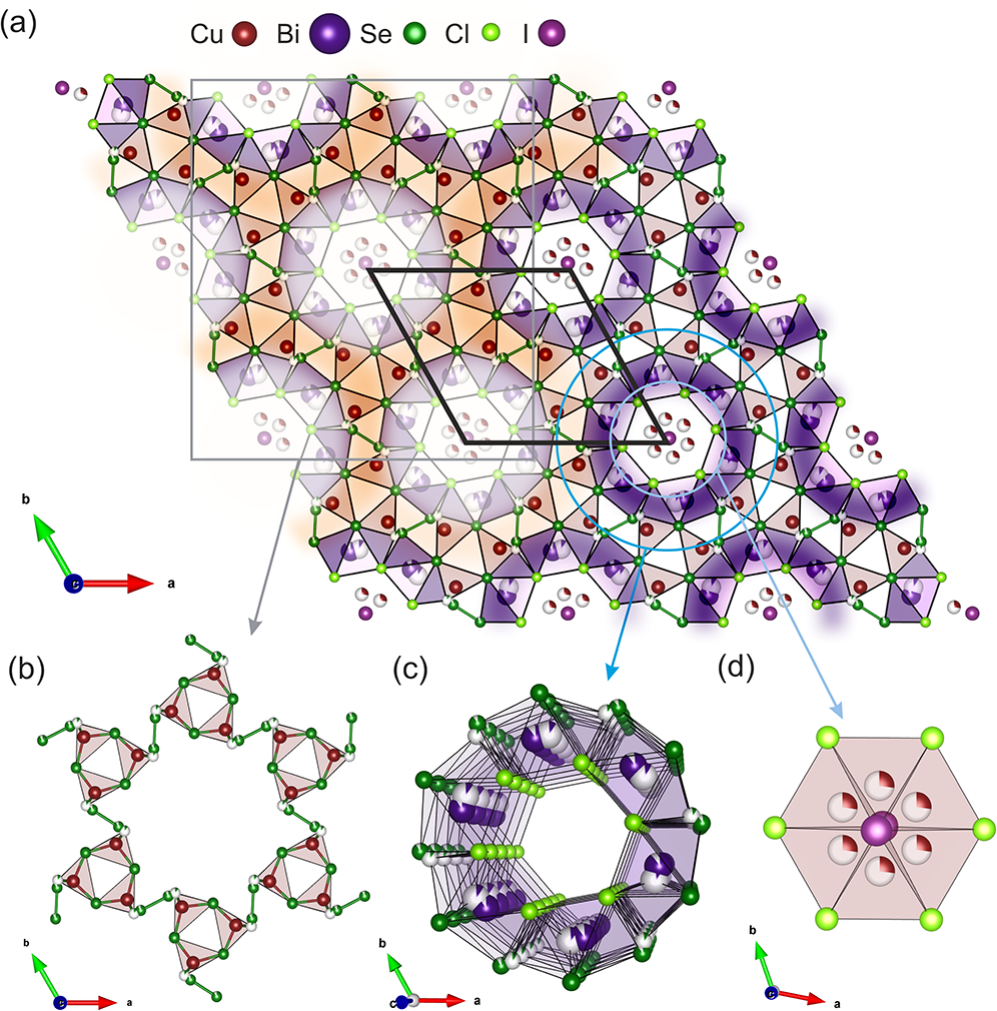
(a) Structure of Cu_7.62_Bi_6_Se_12_Cl_6_I is made up of a network of (b) CuSe_4_ tetrahedra linked by common Se sites and dichalcogenide bridges and (c) cylindrical arrangements of face-sharing Bi–Se–Cl polyhedra, occupied by (d) disordered CuCl_2_I_2_ tetrahedral environments. The black line in (a) represents the unit cell.

There are four anionic species present in Cu_7.62_Bi_6_Se_12_Cl_6_I: Cl^–^, Se^2–^, (Se_2_)^2–^ and I^–^. The arrangement and packing of these anions are depicted in Figure 2. Interestingly, the larger and more polarizable (Se_2_)^2–^ and I^–^ anions form layers (Layer A) separate from the smaller Cl^–^ and Se^2–^ anions (Layer B) (Figure 2(a) and (b)). Layer B consists of arrangements of regular Cl^–^ hexagons (shown in pink) and equilateral triangles of Se^2–^ anions (shown in orange), which, when packed together, generate a distorted kagome net (Figure 2(b)). The arrangement of (Se_2_)^2–^ groups and I^–^ anions in Layer A resembles that of a close-packed layer of spheres, with a large separation of 7.5145(5) Å (equivalent to *a*/2). These layers then stack in an *ABAB* sequence along the *c*-axis (Figure 2(c)), in which the observed packing relates to the hexagonal symmetry of the structure to generate the anion sublattice of Cu_7.62_Bi_6_Se_12_Cl_6_I (Figure 2(d)). The layer stacking places each I^–^ anion between the centroids of superimposed regular Cl^–^_6_ hexagons in both neighboring layers, and the (Se_2_)^2–^ groups between the centroids of superimposed irregular Se_4_Cl_2_ hexagons in neighboring layers to produce the structural motifs displayed in Figure 1(b). This combination of four anionic species in Cu_7.62_Bi_6_Se_12_Cl_6_I generates a complex anionic sublattice, which generates a range of distinct coordination environments available for cation occupancy and thus different types and strengths of bonding, generating considerable structural anisotropy.

**Figure 2:**
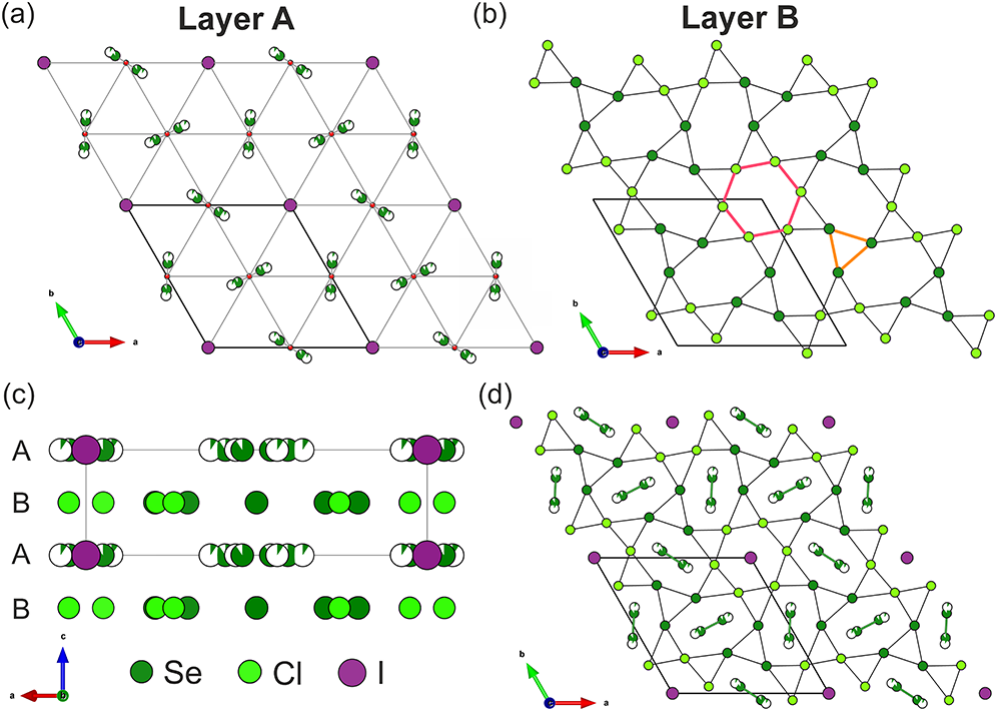
(a) Layer A consists of a regular arrangement of I^–^ anions and Se^2–^/(Se_2_)^2–^ units, depending on whether the Se1 or Se3 site is occupied. (b) Layer B is made up of regular hexagons formed of Cl^–^ anions (outlined in pink) and regular equilateral triangles formed of Se^2–^ anions (outlined in orange), which combine to form a distorted Kagome net. (c) The view along the *b*-axis shows two distinct anionic layers that stack in an *ABAB* arrangement. (d) The view along the *c*-axis of the packing of Se^2–^, (Se_2_)^2–^, Cl^–^ and I^–^ anions in Cu_7.62_Bi_6_Se_12_Cl_6_I.

There are four local cation environments in Cu_7.62_Bi_6_Se_12_Cl_6_I. The Bi^3+^ content is split across two disordered positions, only 0.655(5) Å apart, and forms a five-coordinate square pyramidal (Bi1)­Se_3_Cl_2_, or a seven-coordinate monocapped trigonal prism (Bi2)­Se_3_Cl_4_ environment, depending on the specific site occupancy. The occupancies of the Bi1 and Bi2 sites correlate to those of the neighboring Se1 and Se2 positions, respectively, which determine the local Bi environment, depicted in Figure 3(a)­(i) and (ii).

**Figure 3:**
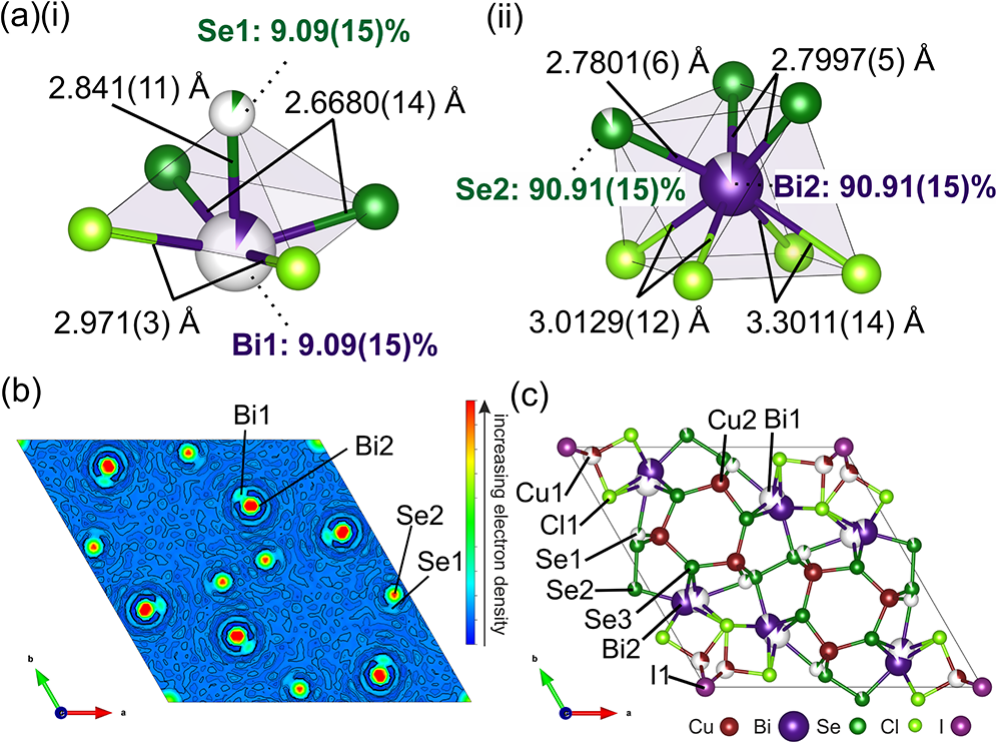
(a) Local environments of Bi^3+^ in Cu_7.62_Bi_6_Se_12_Cl_6_I. Bi^3+^ occupies either (i) a low-occupancy BiSe_3_Cl_2_ square pyramidal environment or (ii) the higher-occupancy seven-coordinate BiSe_3_Cl_4_ local coordination environment. (b) Isosurface slice of electron density in the unit cell of Cu_7.62_Bi_6_Se_12_Cl_6_I, calculated using MEM, with regions of electron density that correlate to the Bi/Se disordered sites labeled. (c) Unit cell of Cu_7.62_Bi_6_Se_12_Cl_6_I viewed along the *c* direction with labeled atomic sites.

The high-occupancy Bi2 site (6*j* Wyckoff position), with an occupancy of 0.9091(15), yields a monocapped trigonal prism with four coordinating Cl^–^ species, two Se^2–^ anions, and one of the Se^–^ in the (Se_2_)^2–^ diselenide pair. The occupancy of this Se atom is also 0.9091(15), meaning that the diselenide bond is present 90.9% of the time in the average structure. On the other hand, Bi1 (6*j* Wyckoff position) has an occupancy of 0.0909(15) and locally forms a square pyramidal coordination environment, in which Bi^3+^ is bonded to two Cl^–^ species and three Se^2–^ anions. Two of these Se anions are fully occupied (Se3), while one has a matching partial occupancy of 0.0909(15) (Se1), which represents a breaking of the diselenide bond in 9.1% of the average structure. All bond lengths are within the expected ranges as reported in the ICSD. The Se–Se distance of 2.3809(11) Å between the two maximally occupied Se2 sites compares well with reported values, consistent with (Se_2_)^2–^ bond lengths from the literature.^36,37^ The distance between the minimally occupied Se1 sites is 3.526(19) Å.

The presence of the disordered Bi1 and Bi2 positions in Cu_7.62_Bi_6_Se_12_Cl_6_I was validated via Maximum Entropy Method (MEM) calculations, as shown in Figure 3(b). The electron density maps depict an isotropic area of electron density positioned on the maximally occupied Bi2 site, and a smaller, distinct region of electron density adjacent to this site that corresponds to the low-occupancy Bi1 site. The distance of 0.655(5) Å between the Bi1 and Bi2 sites is too short to represent the distance between the cation and lone pair.^38^ Anisotropic regions of electron density are also observed at the neighboring Se1/Se2 sites (Figure 3(b)), which are constrained in the structural model to have the same occupancy as the corresponding Bi1/Bi2 sites. This Bi1/Bi2 and Se1/Se2 disorder is further corroborated by the observation of multiple components in the experimentally measured XPS spectra (Figure 4(a)). Two components arise in the Bi 4f core level from the distinct bonding environments (Figure 4(a)); the smaller peak at higher binding energy is attributed to the lower-occupancy Bi1 site, which coordinates with fewer anions. Hence, the core level electrons experience less repulsion, requiring more energy to be ionized, while the larger peak at lower binding energy is associated with the seven-coordinate Bi2 position. Contributions from both Se^2–^ and (Se_2_)^2–^ species in Cu_7.62_Bi_6_Se_12_Cl_6_I were also confirmed via XPS of the Se 3d core level (Figure 4(b)), with the lower binding energy doublet (Se 3d_5/2_: 54.00 eV) corresponding to the Se^2–^ oxidation state,^39^ in good agreement with literature values (Table S5), and the higher binding energy signal (Se 3d_5/2_: 54.85 eV) arising from the (Se_2_)^2–^ oxidation state.^40^ The presence of diselenide bonding in Cu_7.62_Bi_6_Se_12_Cl_6_I is additionally confirmed by Raman spectroscopy (Figure 4(c)), in which a vibrational mode observed at 275 cm^–1^ is assigned to the stretching of the (Se_2_)^2–^ dumbbell unit, as also observed for Cu_7.4_Bi_6_Se_12_Cl_7_.^35^ Diselenide bonds are known to exist in a number of Cu–Bi–Se ternary and quaternary materials, such as Cu_4_Bi_4_Se_9_ (*Pnma*)^37^ Cu_4_BiSe_4_I (*Pnma*)^41^ and Cu_6_BiSe_6_ (*Pnma*).^36^ XPS core level analysis indicates oxidation states of Cu^+^, Bi^3+^, Cl^–^ and I^–^ in Cu_7.62_Bi_6_Se_12_Cl_6_I, with fitting parameters and peak binding energies presented in the Supporting Information S4–S11.

**Figure 4:**
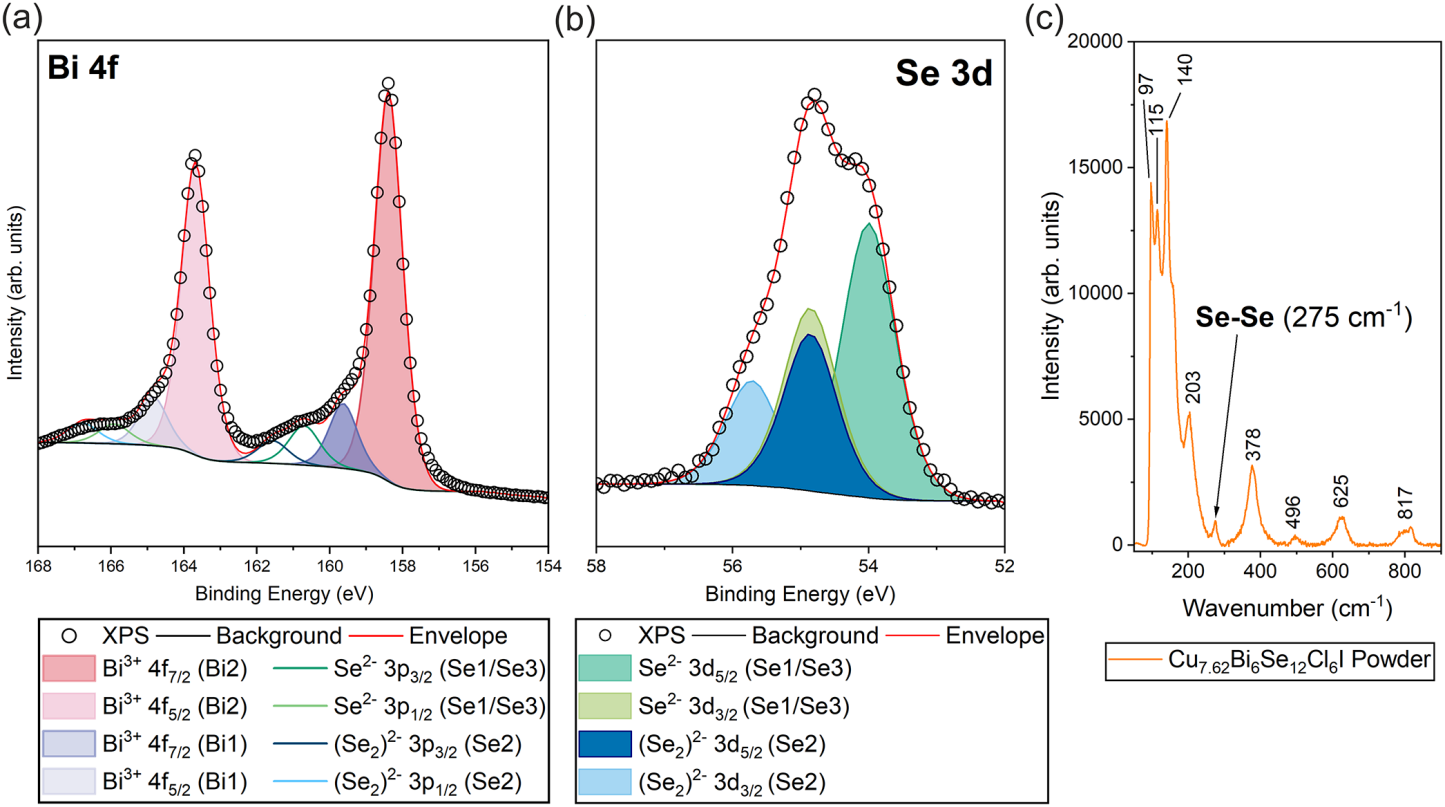
Peak-fitting analysis of the (a) Bi 4f and (b) Se 3d core levels measured using XPS. The presence of diselenide bonds is confirmed via (c) Raman analysis of Cu_7.62_Bi_6_Se_12_Cl_6_I that reveals a distinct Se–Se signature.

Figure 5 shows the two coordination environments of Cu^+^ in Cu_7.62_Bi_6_Se_12_Cl_6_I. The Cu1 position (6*k*), shown in Figure 5(a) and (i), has a Cu^+^ occupancy of 0.270(3) and coordinates two Cl^–^ anions and two I^–^ anions to give the face-shared CuCl_2_I_2_ tetrahedra within the BiSe_3_Cl_4_ tunnels. The Cu2 position (6*k*) is fully occupied, forming CuSe_4_ tetrahedra that connect neighboring BiSe_3_Cl_4_ tunnels (Figure 5(a)­(ii)). These tetrahedra incorporate the Se1 and Se2 sites of the diselenide (Se_2_)^2–^ anions, so the exact local configuration of the tetrahedron depends on the Se1/Se2 site disorder and the correlated occupancy of either the Bi1 or Bi2 positions in the adjacent environment. Adjacent CuSe_4_ tetrahedra are linked in 90.9% of the average structure via occupancy of the Se2 position, as shown in Figure 5(b)­(i) and (ii).

**Figure 5:**
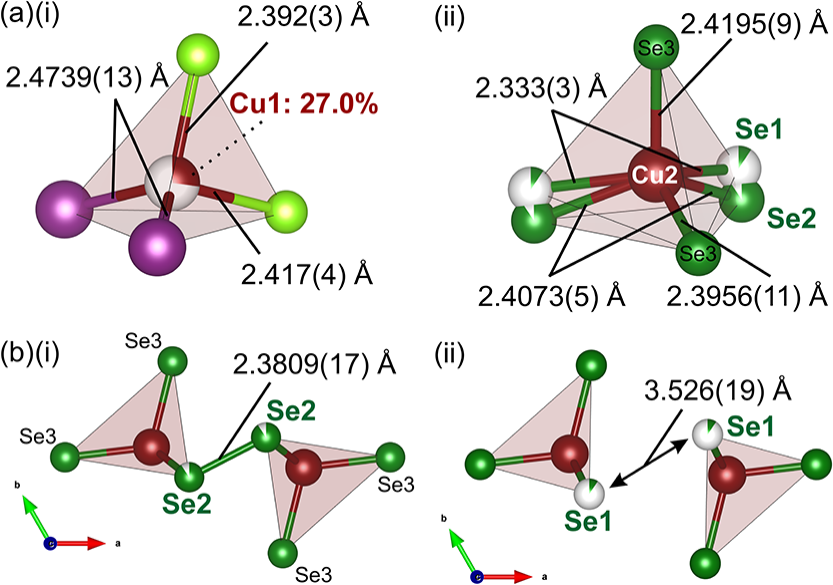
(a) The two Cu^+^ environments in Cu_7.62_Bi_6_Se_12_Cl_6_I are (i) the partially occupied Cu1 site, which forms a CuCl_2_I_2_ tetrahedral environment, and (ii) the fully occupied Cu2 site, which forms a CuSe_4_ tetrahedron bonded to either the Se1 or Se2 species, depending on the coordination of the neighboring Bi environment. All bonding environments are labeled with bond lengths and site occupancies. (b) Depictions of the Cu2 environment: (i) when bonded to the Se2 species, which forms diselenide bonds between adjacent Se2 sites, and (ii) when bonded to the Se1 species, which are too far apart from each other for diselenide bonds to form.

Though Cu_7.62_Bi_6_Se_12_Cl_6_I is isostructural with Cu_7.4_Bi_6_Se_12_Cl_7_,^35^ it is the first experimentally realized material in the quinary Cu–Bi–Se–Cl–I phase space. Other materials in the lower-dimensional Cu–Bi–Se–Cl space include CuBiSeCl_2_ ^18^ and Cu_3.58(1)_Bi_4.42(1)_Se_6.84(2)_Cl_3.16(2)_.^42^ The structures of Cu_7.62_Bi_6_Se_12_Cl_6_I and Cu_7.4_Bi_6_Se_12_Cl_7_ are compared in Figure S12. The lattice parameters of Cu_7.62_Bi_6_Se_12_Cl_6_I (*a = b =* 15.0289(1) Å, *c =* 4.0145(1) Å) are larger than Cu_7.4_Bi_6_Se_12_Cl_7_ (*a = b =* 14.972(3) Å, *c =* 3.9954(6) Å) due to the incorporation of the larger I^–^ anion (*r*_Cl_^–^ = 1.81 Å vs *r*_I_^–^ = 2.2 Å) on the 1*a* Wyckoff position,^43^ with the preferential occupancy of this site understood through the consideration of anion packing described above. There is an associated increase in the CuCl_2_I_2_ tetrahedral volume to 7.284 Å^3^ in Cu_7.62_Bi_6_Se_12_Cl_6_I (compared to 7.194 Å^3^ for the CuCl_4_ environment in Cu_7.4_Bi_6_Se_12_Cl_7_), in which the larger I^–^ acts to reduce the degree of Cu^+^ site disorder onto a single site with 0.270(3) occupancy, compared to three distinct sites each with 0.059 occupancy in Cu_7.4_Bi_6_Se_12_Cl_7_. Increased Cu^+^ localization was also observed between Cu_1.5_Bi_2.64_S_3.42_Br_2.58_ (*C*2/*m*) and Cu_2.31_Bi_5_Se_8.31_I_0.69_ (*C*2/*m*),^44^ likely resulting from the inclusion of the larger Se^2–^ and I^–^ anions in the latter compound.

Phase-pure powder samples of Cu_7.62_Bi_6_Se_12_Cl_6_I were synthesized (S13–S14) to facilitate property measurements from a bulk sample. Needle-like crystallites are observed via TEM (Figure S15), while a homogeneous elemental distribution is confirmed through mapping via SEM-EDX measurements (Figure S16). A measured composition of Cu_7(1)_Bi_5.2(5)_Se_11.5(9)_Cl_6.4(6)_I_1.0(3)_ is obtained from EDX (Figure S17) and WDX measurements, in good agreement with the refined composition from the single crystal solution. There is generally good agreement between the two compositions, with all elemental compositions from EDX/WDX analysis within error of the single-crystal values, except for Bi. The Bi content measured by EDX is within 1.5σ of the refined composition from the single-crystal solution but is slightly lower than expected, likely due to the overlapping contribution of the Cl peak.

We also performed air stability measurements on Cu_7.62_Bi_6_Se_12_Cl_6_I, which show that this material exhibits stability under ambient conditions for up to 2 weeks (S18).

## 4 Optical Properties and Band Alignments

UV–vis spectrometry was used to determine the band gap of the material, as shown in Figure 6 (a). Tauc analysis of the Kubelka–Munk-converted diffuse reflectance data reveals a low direct band gap of 0.94(5) eV. The indirect band gap is measured to be 0.88(3) eV (Figure S19), though it is likely that the direct band gap dominates the optical absorption due to the proximity of the direct and indirect band gaps. This is because it does not require a phonon-assisted transition, as in the case of an indirect band gap transition. The band gap of Cu_7.62_Bi_6_Se_12_Cl_6_I is lower than those of other 5*s* ^2^- or 6*s* ^2^-containing chalcogenide and halide-chalcogenide materials, namely Sb_2_Se_3_,^45^ CuSbS_2_,^46^ Cu_3_BiS_3_,^47^ and CuBiSeCl_2_, which all exhibit gaps in the range of 1.18–1.55 eV. The band alignments of these materials are compared against that of Cu_7.62_Bi_6_Se_12_Cl_6_I in Figure 6 (b). By measuring very low power XPS to capture the secondary electron cutoff (S20–S21), the ionization potential for Cu_7.62_Bi_6_Se_12_Cl_6_I was determined to be 4.27 eV (S22).

**Figure 6:**
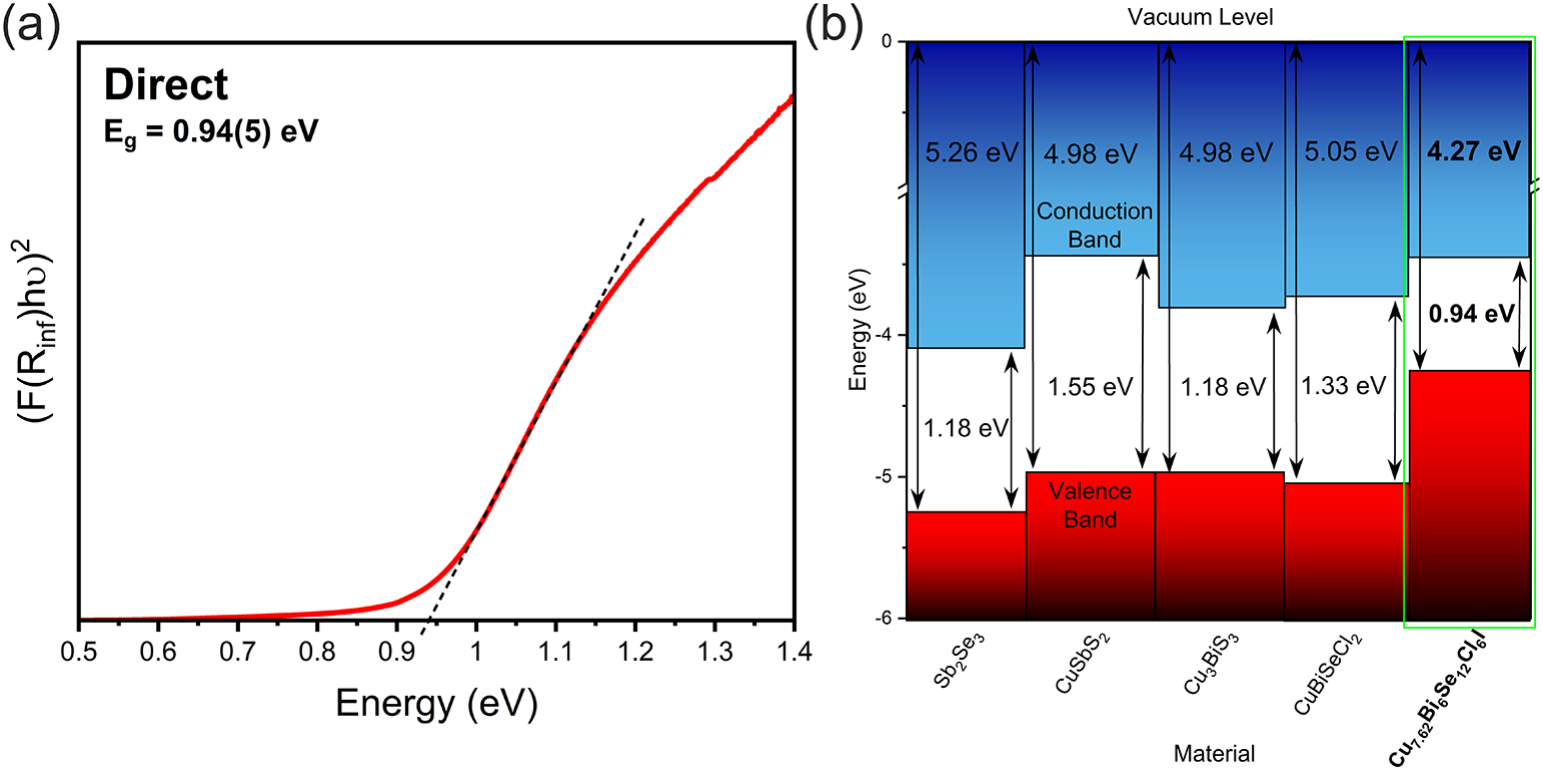
(a) Direct band gap determined from Tauc analysis of UV–vis spectrometry data measured on powder samples of Cu_7.62_Bi_6_Se_12_Cl_6_I. (b) Band alignments of Cu_7.62_Bi_6_Se_12_Cl_6_I determined using XPS measurement of the ionization potential (IP) and the band gap measured using UV–vis spectrometry. Band alignments are compared with those determined for Sb_2_Se_3_,^45^ CuSbS_2_,^46^ Cu_3_BiS_3_,^47^ and CuBiSeCl_2_.^18^ At present, the band alignments for CuBiSe_2_ have not been characterized; hence, CuSbS_2_ is chosen as a substitute comparator owing to its similar chemistry.

Red-shifting of the band gap via iodine substitution to alter the orbital contributions to the valence and conduction bands is a well-known phenomenon observed in a range of inorganic materials, including halide-chalcogenide compounds.^48,49^ Interestingly, this appears to be the case even though I^–^ occupies only a single site within the CuCl_2_I_2_ tetrahedra and does not permeate the whole structure, indicating that the CuCl_2_I_2_ coordination environment contributes significantly to the overall electronic structure of Cu_7.62_Bi_6_Se_12_Cl_6_I. The lower IP of Cu_7.62_Bi_6_Se_12_Cl_6_I can be understood, in the absence of density of state calculations (precluded by the presence of disorder), by considering the electronic makeup of related materials. It has been shown that iodide *p* orbitals have a commanding effect on the electronic structure of the valence band of a range of materials.^50–53^ This has previously been observed in the calculated band alignments of BiOX (X = F^–^, Cl^–^, Br^–^, I^–^) as studied by Ganose et al.^54^ Increasing the radius of the halide anion from F^–^ to I^–^ reduces the IP by repositioning the relative energy levels, lowering the IP from 8.23 eV in BiOF to 7.03 eV in BiOI. It is therefore likely that the relative energy levels of I 5*p* states in the valence band result in the reduction of IP in Cu_7.62_Bi_6_Se_12_Cl_6_I.

The observed change of band alignments in Cu_7.62_Bi_6_Se_12_Cl_6_I infers the enhanced control available over the electronic structure that is possible through anionic substitution of Cl^–^ for I^–^. The narrow band gap, achieved via the introduction of a fourth anion, means that Cu_7.62_Bi_6_Se_12_Cl_6_I absorbs light in the ideal region for potential application as the bottom layer in a 2-terminal photovoltaic tandem device.^55,56^ However, we note that the extensive cation disorder may be a limiting factor for open-circuit voltage in solar cells, which has already been demonstrated in Cu_2_ZnSnS_4_ (CZTS).^52,57^ Access to a range of controllable band gaps could be made possible by further compositional tuning of the anion stoichiometry in Cu_7.62_Bi_6_Se_12_Cl_6_I.

## 5 Thermal Transport Properties

Thermal conductivity (κ) and heat capacity (*C*_p_) data were measured between 2 and 293 K from a dense pellet (∼93% density) of Cu_7.62_Bi_6_Se_12_Cl_6_I (Figure 7). Cu_7.62_Bi_6_Se_12_Cl_6_I has a low thermal conductivity of 0.25(2) W K^–1^ m^–1^ at room temperature and exhibits a broad low-temperature maximum of 0.4483(4) W K^–1^ m^–1^ at 43.3 K (Figure 7(a)), which is generally indicative of phonon-crystal thermal transport behavior, in which heat is transported via propagating phonons. The low-temperature peak likely results from the effect of scattering due to point defects attributed to the considerable structural disorder in the material, which increases phonon scattering events and reduces the phonon mean free path.^60^

**Figure 7:**
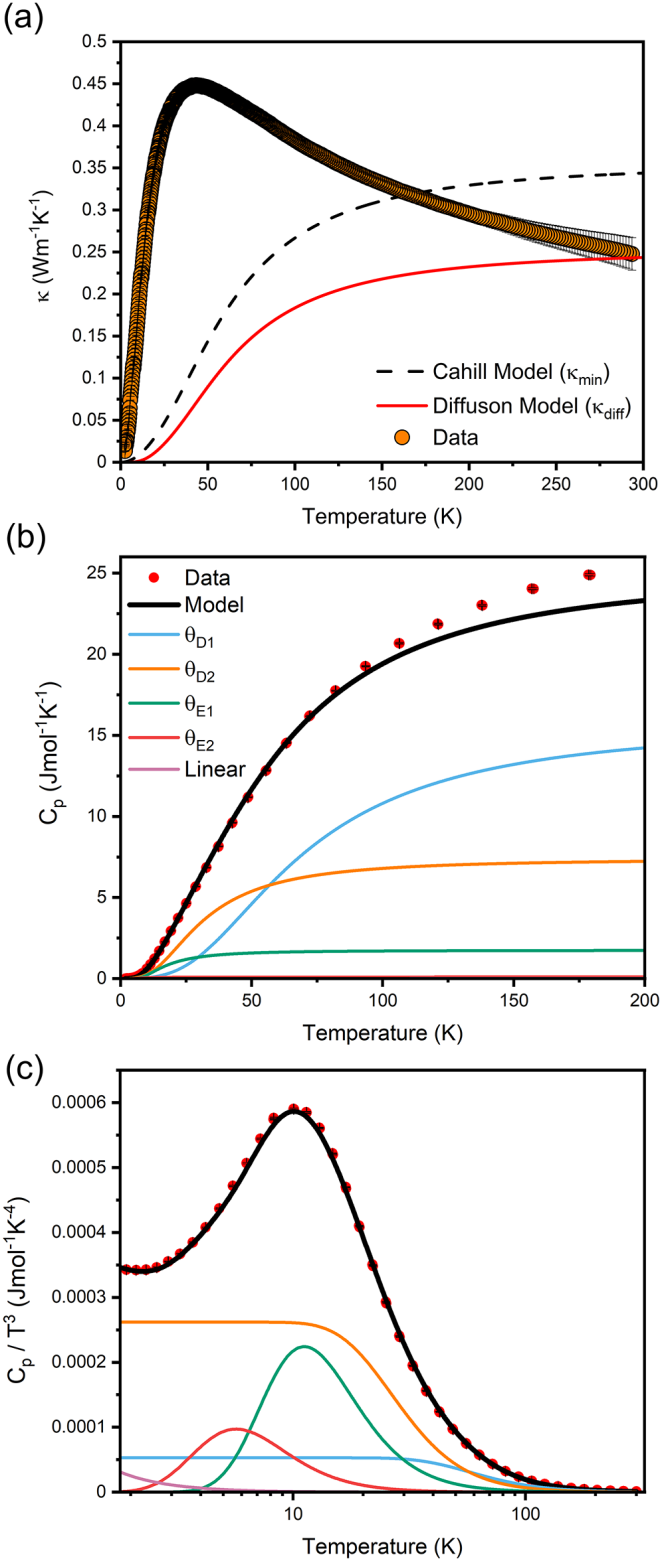
(a) Thermal conductivity data measured in the 2–293 K temperature range on a dense pellet of Cu_7.62_Bi_6_Se_12_Cl_6_I with associated Cahill (κ_min_)^58^ and diffuson (κ_diff_)^59^ models. The measurement was performed parallel to the pressing direction. Fitted heat capacity data of Cu_7.62_Bi_6_Se_12_Cl_6_I are shown as (b) C_p_ and (c) C_p_/T^3^ with individual Debye, Einstein, and linear (γ) components shown.

The heat capacity data of Cu_7.62_Bi_6_Se_12_Cl_6_I were modeled, as shown in Figure 7(b) and (c), using a combination of Debye and Einstein temperatures, which are summarized in Table 1. Two Debye terms, two Einstein terms and a linear term are required to accurately reproduce the experimental data and the excess specific heat observed at low temperatures (Figure S23 and Table S24). Distinct Debye temperatures can be indicative of bonding anisotropy in the structure, whereas Einstein contributions represent localized oscillators. Thus, the need for both terms in the modeling suggests that the low thermal conductivity of Cu_7.62_Bi_6_Se_12_Cl_6_I arises from a combination of anisotropic bonding and localized structural vibrations, reflecting the structural complexity of the material.

**Table 1:** Debye and Einstein Temperatures Obtained from Modeling of Experimental Heat Capacity Data Measured on Cu_7.62_Bi_6_Se_12_Cl_6_I

Material	θ_D1_ (K)	θ_D2_ (K)	θ_E1_ (K)	θ_E2_ (K)	γ (J mol^–1^ K^–2^)
Cu_7.62_Bi_6_Se_12_Cl_6_I	285(2)	130(1)	55(1)	28(1)	0.0001(1)

Using the Debye temperatures extracted from the heat capacity data, two theoretical frameworks developed by Cahill^58^ (κ_min_) and Agne (κ_diff_)^59^ were used to approximate the minimum thermal conductivity of Cu_7.62_Bi_6_Se_12_Cl_6_I (S23); these results are depicted in Figure 7(a). The κ_diff_ model of Agne et al., which accounts for diffuson-mediated thermal transport in the material, more accurately reflects the room temperature thermal conductivity than the model developed by Cahill et al.; however, it does not capture the behavior observed at low temperatures. This suggests that diffuson-mediated thermal transport is the dominant process of heat transfer in Cu_7.62_Bi_6_Se_12_Cl_6_I at higher temperatures, while scattering processes related to propagons are more dominant at low temperatures, accounting for the observed broad peak. Numerous studies^61–63^ have found that the diffuson model is more successful than the propagon model at describing the thermal conductivity of materials with complex structures and considerable disorder. The structure of Cu_7.62_Bi_6_Se_12_Cl_6_I comprises significant bonding anisotropy alongside extensive Bi^3+^, (Se_2_)^2–^ and Cu^+^ site disorder; thus, the diffuson model, which considers a random walk of vibrations through the lattice, is a better approximation to the vibrational behavior in the material.

Bonding anisotropy and structural anharmonicity are prominent characteristics of materials that exhibit ultralow thermal conductivities; disruption to bonding within a structure reduces the speed of sound and the transport of heat through the material. The presence of two distinct Debye temperatures aligns with the complex hierarchy of bonding anisotropy and dimensionality observed in the structure of Cu_7.62_Bi_6_Se_12_Cl_6_I and reflects the combination of multiple anionic species with different properties, such as size, charge, and polarizability. For example, Cu^+^ coordinates with all four types of anion (Se^2–^ and (Se_2_)^2–^, and Cl^–^ and I^–^) across the two distinct positions that it occupies. The spatial separation of these anion types, and thus their bonding characteristics, into weaker halide-only (CuCl_2_I_2_) and stronger selenide-only (CuSe_4_) Cu^+^ environments itself will increase phonon scattering and reduce the thermal conductivity.^64–66^

Heat transfer in Cu_7.62_Bi_6_Se_12_Cl_6_I is further disrupted by two separate Einstein contributions observed in the experimental heat capacity at low temperatures (Figure 7c); these are θ_E1_ = 55 K and θ_E2_ = 28 K. These contributions represent those of localized oscillators, each with a narrow range of vibrational frequencies, making them nonpropagating. The lower frequency θ_E2_ term is likely attributed to a combination of factors associated with heavier species in the structure, namely the disordered Bi^3+^ positions and also the I^–^ site, which exhibits the largest atomic displacement parameter (*U*_iso_ = 0.0486(3) Å^2^) of any species in Cu_7.62_Bi_6_Se_12_Cl_6_I (Table 2), with significant anisotropy. The higher θ_E1_ frequency is associated with the lighter-weight disordered Cu^+^ position (Cu1) that coordinates the I^–^ which also displays a large displacement parameter of 0.0219(10) Å^2^. The large displacement parameters observed for these neighboring positions reflect the weak Cu–I bonding expected between the two species, as was shown in CuBiI_4_.^67^ Cu^+^ is shown to behave as a localized oscillator in related materials, including Cu_1.6_Bi_4.8_S_8_, which also contains several weakly bonded, disordered Cu^+^ environments within a Bi–S “host framework”,^68^ while large iodine atomic displacement parameters are commonly observed in halide perovskites such as methylammonium lead iodide^69^ and CsPbI_3_, which arise from anharmonicity of the Pb–I bond.^70^

**Table 2:** Equivalent Isotropic Displacement Parameters (*U*_eq_) and Anisotropic Displacement Parameters (Å^2^) for Cu_7.62_Bi_6_Se_12_Cl_6_I from Refinement against SCXRD Data at 100 K

Atom	*U*_eq_	*U*_11_	*U*_22_	*U*_33_	*U*_23_	U_13_	U_12_
Cu1	0.0219(10)	0.0108(13)	0.0178(14)	0.0303(18)	0	0	0.0021(10)
Cu2	0.01055(15)	0.0091(3)	0.0086(3)	0.0140(4)	0	0	0.0044(2)
Bi1	0.0047(8)	0.00921(13)	0.00537(15)	0.00841(15)	0	0	0.00363(9)
Bi2	0.00767(11)	0.00921(13)	0.00537(15)	0.00841(15)	0	0	0.00363(9)
Se1	0.0045(16)	0.0069(3)	0.0059(3)	0.0087(3)	0	0	0.0040(2)
Se2	0.00682(15)	0.0069(3)	0.0059(3)	0.0087(3)	0	0	0.0040(2)
Se3	0.00712(13)	0.0076(2)	0.0046(2)	0.0090(3)	0	0	0.00298(17)
Cl1	0.0120(3)	0.0096(5)	0.0114(6)	0.0182(7)	0	0	0.0075(5)
I1	0.0486(3)	0.0637(5)	0.0637(5)	0.0185(6)	0	0	0.0318(3)

The increased structural complexity of Cu_7.62_Bi_6_Se_12_Cl_6_I leads to a synergy of several distinct structural features that together suppress heat transport within the material, including: (1) a hierarchy of distinct bond strengths; (2) heavy and stereochemically active lone-pair cations such as Bi^3+^; (3) the presence of localized oscillators; and (4) extensive site disorder that likely correlates at local length scales between neighboring environments. These structural characteristics, most of which result from the direct combination of four different anion types, contribute cooperatively to determine the low thermal conductivity of 0.25(2) W K^–1^ m^–1^ in Cu_7.62_Bi_6_Se_12_Cl_6_I.

## 6 Conclusions

The synthesis, structure, optoelectronic, and thermal transport properties of Cu_7.62_Bi_6_Se_12_Cl_6_I, the first quinary material in the Cu–Bi–Se–Cl–I phase space, are reported. The impact of combining four distinct anion types becomes evident from the multiple unique and complex structural motifs that result from the cation occupation of environments generated from anion packing. This complexity leads to a broad variety of bonding that defines extensive structural anisotropy, disorder, localized oscillator effects, and anharmonicity, which significantly reduce phonon transport within the material to achieve the measured low thermal conductivity. The low band gap of 0.94(2) eV indicates potential applications in optoelectronics, and the structural anisotropy makes it a candidate material for thin-film development. These baseline properties across multiple potential application areas offer opportunities for further property modification via the control of structure through composition, e.g., both the thermal and electronic transport behavior could be further tuned and optimized by influencing the degree of site disorder and through the introduction of potential site mixing on cation and/or anion positions.

## Supplementary Material



## Data Availability

The data that support the findings of this research are openly available via the University of Liverpool data repository: 10.17638/datacat.liverpool.ac.uk/3063.

## Notes

EDX from exploratory synthesis; crystal structure information (structural description, oxidation state analysis, Powder X-ray Diffraction); compositional analysis (TEM, SEM-EDX); environmental stability (PXRD); electronic structure: indirect band gap Tauc plot, band alignment determination method; thermal properties (specific heat capacity models); and additional references (PDF)
