# Supplementary material for: Cu7.62Bi6Se12Cl6I: Discovery of a Low Band Gap, Low Thermal Conductivity Mixed-Anion Material
Source: Chem Mater. 2026 Feb 24;38(7):3286–96. doi: 10.1021/acs.chemmater.5c02919 (PMC13084983; doi:10.1021/acs.chemmater.5c02919)
Supplement: Supplementary file 1 [file cm5c02919_si_001.pdf]

Supplementary Information

$Cu_{7.62}Bi_6Se_{12}Cl_6I$: Discovery of a Low Band Gap, Low Thermal Conductivity Mixed Anion Material

Cara J. Hawkins[a], Batoul Almoussawi[a], Jan P. Scheifers[a,b], Manel Sonni[a], Aeshah A. Almushawwah[c, d], Troy D. Manning[a], Marco Zanella[a], Craig M. Robertson[a], Luke M. Daniels[a], Tim D. Veal[c], John B. Claridge[a,b] and Matthew J. Rosseinsky[a,b]*

a. Department of Chemistry, Materials Innovation Factory, University of Liverpool, 51 Oxford Street, Liverpool, L7 3NY, U.K.

b. Leverhulme Research Centre for Functional Materials Design, Materials Innovation Factory, University of Liverpool, 51 Oxford Street, Liverpool, L7 3NY, U. K.

c. Stephenson Institute for Renewable Energy and Department of Physics, University of Liverpool, Liverpool L69 7ZF, U.K.

d. Department of Physics, Faculty of Science and Humanities in Al-Dawadmi, Shaqra University, Shaqra 11911, Saudi Arabia.

* Corresponding Author: M.J.Rosseinsky@liverpool.ac.uk

The Supplementary Information is divided into the following sections

1. **Exploratory Synthesis**
2. **Crystal Structure**
   i. **Structural Description**
   ii. **Oxidation State Analysis**
   iii. **Powder X-Ray Diffraction**
3. **Compositional Analysis**
4. **Environmental Stability**
5. **Electronic Structure**
6. **Thermal Properties**

Exploratory Synthesis

S0. SC-EDX data analysis of the relative amounts of the cations Cu, Bi, and Zn, measured on a single crystal of $Cu_{7.62}Bi_6Se_{12}Cl_6I$ screened from initial exploratory reactions, which included a $ZnCl_2$ precursor. The red point indicates the target cation stoichiometry.

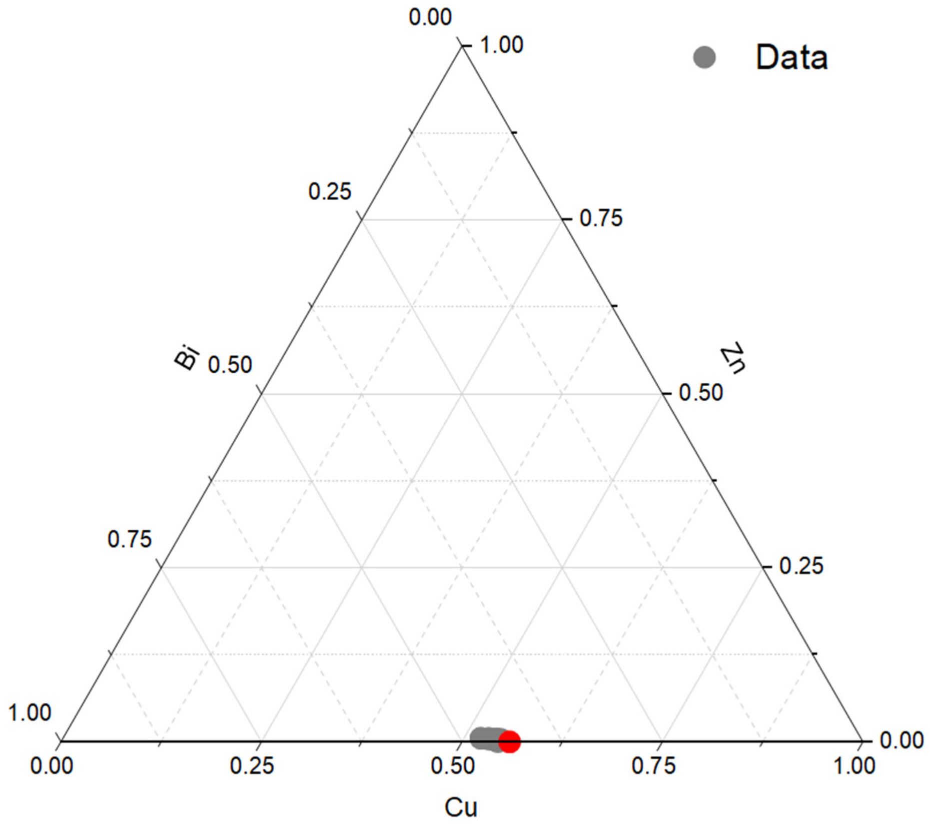

Crystal Structure

I. Structural Description

S1. Single Crystal data, data collection and structure refinement parameters of $Cu_{7.62}Bi_6Se_{12}Cl_6I$

**Empirical formula**	**$Cu_{7.62}Bi_6Se_{12}Cl_6I$**
Molecular weight (g. mol^{-1})	3025.17
Temperature (K)	100
Symmetry	*Hexagonal*
Space group	*P*6/*m* (175)
Unit cell dimensions (Å)	*a* = 15.0289(1)<br>*b* = 15.0289(1)<br>*c* = 4.0145(1)
Volume (Å^3)	785.27(2)
Z	1
**Data Collection**	
λ (Å)	0.6889
Calculated density (g cm^{-3})	6.397
Crystal shape	Platelet
Crystal dimensions (mm^3)	0.06 × 0.03 × 0.02
Color	Black
Absorption correction	Empirical
θ (min−max) (°)	3.032-50.912
μ (mm^{-1})	49.637
F(000)	1282.0
Reciprocal space recording	$-18 \leq h \leq 18$,<br>$-18 \leq k \leq 18$,<br>$-5 \leq l \leq 5$
No. of measured reflections	10895
No. of independent reflections	613 [R_{int} = 0.0495, R_{sigma} = 0.0163]
**Refinement**	
Data/restraints/parameters	613/0/48
Refinement method	Full-matrix least-squares on F^2
Weighting scheme	
$R_1(F)$ [$I > 2\sigma(I)$] / $R_1(F^2)$ (all data, %)	0.0128 / 0.0128
wR_2 (F^2) [$I > 2\sigma(I)$] / wR_2 (F^2) (all data, %)	0.0290 / 0.0290
Goodness of Fit	1.180
Max/Min residual electronic density (e^-/Å^3)	0.59/-1.29

S2. Fractional Atomic Coordinates and Equivalent Isotropic Displacement Parameters (Å^2) for $Cu_{7.62}Bi_6Se_{12}Cl_6I$. U_{eq} is defined as 1/3 of the trace of the orthogonalised U_{ij} tensor.

**Atom**	***Wyck.***	***x***	***y***	***z***	**U_{eq}**	**Occ.**
Cu1	6*k*	0.89107(18)	0.9266(2)	1/2	0.0219(10)	0.270(3)
Cu2	6*k*	0.47847(5)	0.84954(5)	1/2	0.01055(15)	1.0
Bi1	6*j*	0.63390(16)	0.7840(2)	0	0.0047(8)	0.0909(15)
Bi2	6*j*	0.64663(2)	0.74823(2)	0	0.00767(11)	0.9091(15)
Se1	6*j*	0.5606(5)	0.9254(5)	0	0.0045(16)	0.0909(15)
Se2	6*j*	0.54191(4)	0.95055(5)	0	0.00682(15)	0.9091(15)
Se3	6*k*	0.49997(4)	0.70198(3)	1/2	0.00712(13)	1.0
Cl1	6*k*	0.79343(9)	0.74294(9)	1/2	0.0120(3)	1.0
I1	1*a*	0	0	0	0.0486(3)	1.0

S3. Bond Lengths for $Cu_{7.62}Bi_6Se_{12}Cl_6I$.

Atom	Atom	Length/Å	Atom	Atom	Length/Å
Bi2	Se3[1]	2.7997(4)	I1	Cu1[11]	2.4738(14)
Bi2	Se3	2.7996(4)	I1	Cu1[12]	2.4738(14)
Bi2	Se2[2]	2.7802(6)	I1	Cu1[13]	2.4738(14)
Bi2	Cl1[1]	3.0131(9)	Se2	Se2[14]	2.3808(16)
Bi2	Cl1	3.0130(9)	Se2	Cu2[4]	2.4074(5)
Se3	Cu2	2.3957(7)	Se2	Cu2	2.4073(5)
Se3	Cu2[3]	2.4195(7)	Cu2	Se1	2.333(3)
Se3	Bi1	2.6681(14)	Cu2	Se1[1]	2.333(3)
Se3	Bi1[4]	2.6682(14)	Cl1	Cu1	2.392(3)
I1	Cu1[5]	2.4738(14)	Cl1	Cu1[2]	2.417(3)
I1	Cu1[2]	2.4738(14)	Cl1	Bi1[15]	2.971(2)
I1	Cu1[6]	2.4738(14)	Cl1	Bi1[2]	2.971(2)
I1	Cu1	2.4739(14)	Cu1	Cu1[7]	1.446(2)
I1	Cu1[7]	2.4738(14)	Cu1	Cu1[13]	2.505(4)
I1	Cu1[8]	2.4738(14)	Cu1	Cu1[12]	2.892(5)
I1	Cu1[4]	2.4738(14)	Cu1	Cu1[2]	1.446(2)
I1	Cu1[9]	2.4738(14)	Cu1	Cu1[6]	2.505(4)
I1	Cu1[10]	2.4738(14)	Se1	Bi1[4]	2.840(10)
Bi2	Se3[1]	2.7997(4)	I1	Cu1[11]	2.4738(14)
Bi2	Se3	2.7996(4)	I1	Cu1[12]	2.4738(14)

[1]+X,+Y,1+Z; [2]1-Y+X,+X,1-Z; [3]+Y-X,1-X,+Z; [4]+X,+Y,-1+Z; [5]2-X,2-Y,1-Z; [6]+Y,1-X+Y,-Z; [7]2-Y,1+X-Y,-1+Z; [8]1+Y-X,2-X,-1+Z; [9]+Y,1-X+Y,1-Z; [10]1+Y-X,2-X,+Z; [11]2-Y,1+X-Y,+Z; [12]1-Y+X,+X,-Z; [13]2-X,2-Y,-Z; [14]1-X,2-Y,-Z; [15]1-Y+X,+X,2-Z

II. Oxidation State Analysis

XPS was used to confirm the oxidation states of all species in $Cu_{7.62}Bi_6Se_{12}Cl_6I$ through comparison with literature reference values. Where possible, Full Width at Half Maximum (FWHM) and energy uncertainty values have been included from literature.

S4. Binding energies and FWHM values determined from fitting the Bi 4f core level measured by XPS on $Cu_{7.62}Bi_6Se_{12}Cl_6I$, compared with values determined from literature.

Bi 4f	Experimental		Literature	
Orbital	**Binding Energy (eV)**	**FWHM (eV)**	**Binding Energy (eV)**	**FWHM (eV)**
**Bi^{3+} $4f_{7/2}$ (Bi2)**	158.39	0.92	158.20 [1]	/
**Bi^{3+} $4f_{5/2}$ (Bi2)**	163.68	0.92	163.40 [1]	/
**Bi^{3+} $4f_{7/2}$ (Bi1)**	159.62	0.92	/	/
**Bi^{3+} $4f_{5/2}$ (Bi1)**	164.83	0.92	/	/
**Se^{2-} $3p_{3/2}$ (Se1)**	160.70	1.00	160.70 [2]	/
**Se^{2-} $3p_{1/2}$ (Se1)**	165.91	1.00	166.20 [3]	/
**$(Se_2)^{2-}$ $3p_{3/2}$ (Se3)**	161.56	1.00	/	/
**$(Se_2)^{2-}$ $3p_{1/2}$ (Se3)**	166.77	1.00	/	/

S5. Binding energies and FWHM values determined from fitting the Se 3d core level measured by XPS on $Cu_{7.62}Bi_6Se_{12}Cl_6I$, compared with values determined from literature.

Se 3d	Experimental		Literature	
Orbital	**Binding Energy (eV)**	**FWHM (eV)**	**Binding Energy (eV)**	**FWHM (eV)**
**Se^{2-} $3d_{5/2}$**	54.00	0.93	54.30[4]	/
**Se^{2-} $3d_{3/2}$**	54.85	0.93	55.00[4]	/
**$(Se)_2^{2-}$ $3d_{5/2}$**	54.85	0.93	54.4[5]	/
**$(Se)_2^{2-}$ $3d_{3/2}$**	55.72	0.93	55.3[5]	/

S6. XPS analysis of Cu 2p core levels measured by XPS on $Cu_{7.62}Bi_6Se_{12}Cl_6I$.

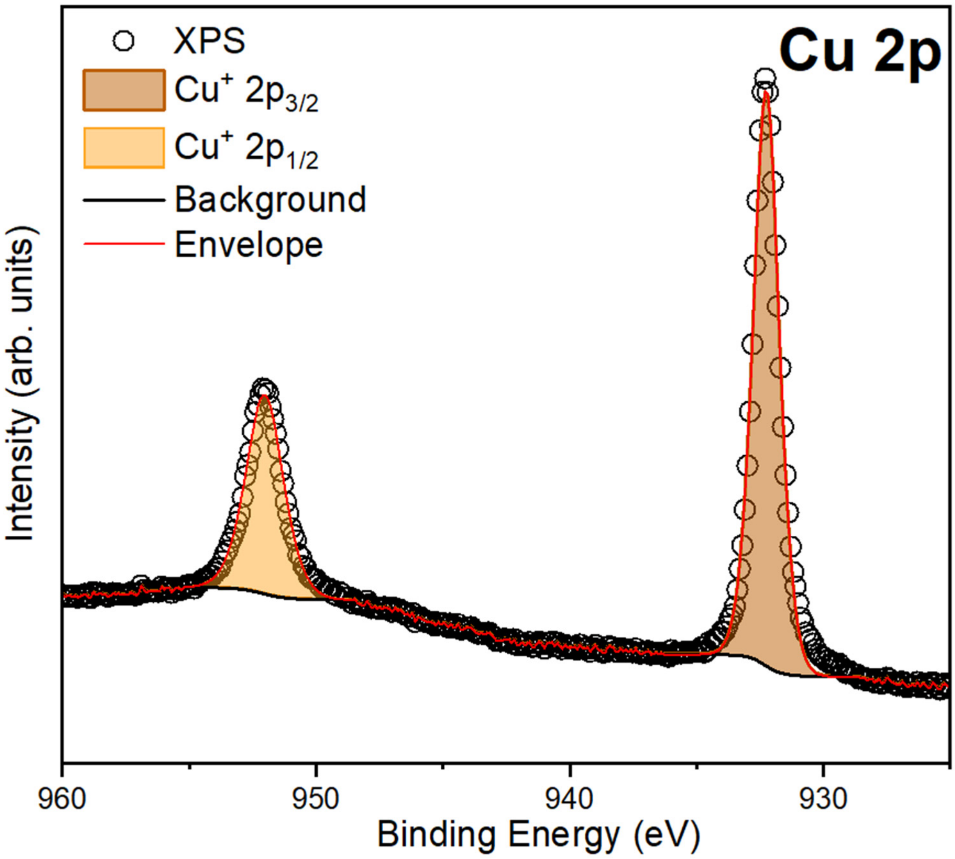

S7. Table of Cu 2p Binding Energies / Fitting Parameters

Cu 2p	Experimental		Literature	
Orbital	**Binding Energy (eV)**	**FWHM (eV)**	**Binding Energy (eV)**	**FWHM (eV)**
**Cu^+ $2p_{3/2}$**	932.25	1.20	932.49 [6]	0.99
**Cu^+ $2p_{1/2}$**	952.02	1.72	952.31 [6]	1.41

S8. XPS Analysis of Cl 2p core levels

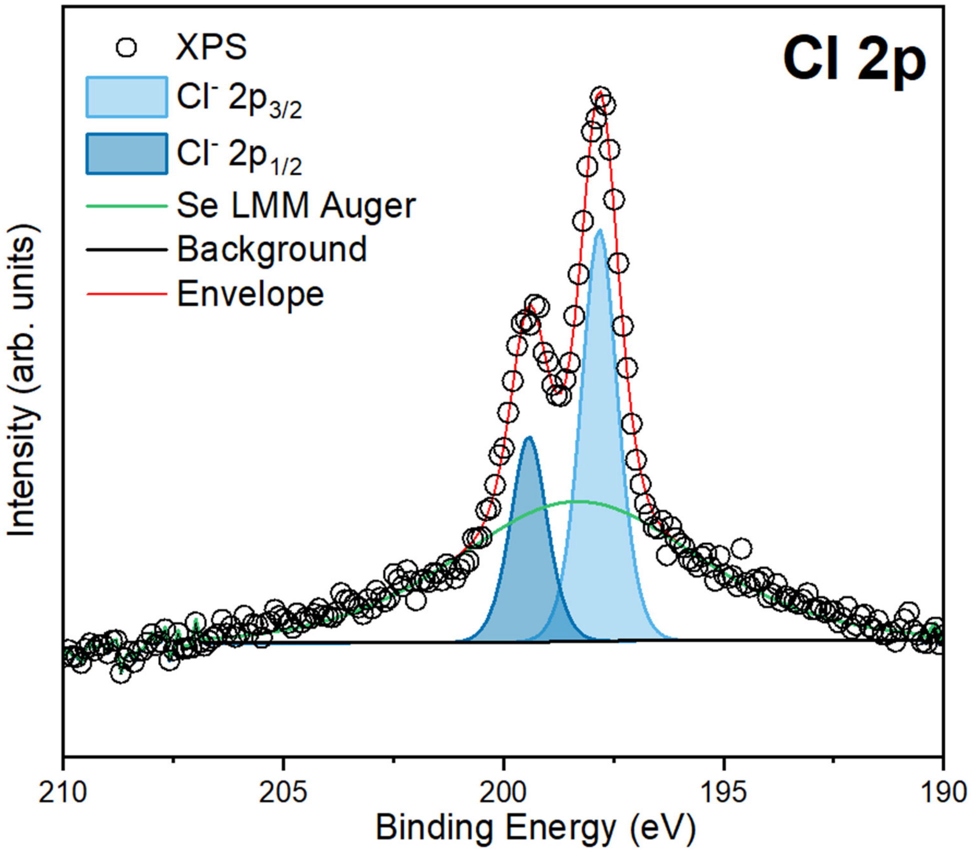

S9. Table of Cl 2p Binding Energies / Fitting Parameters

Cl 2p	Experimental		Literature	
Orbital	**Binding Energy (eV)**	**FWHM (eV)**	**Binding Energy (eV)**	**FWHM (eV)**
**$Cl^- 2p_{3/2}$**	197.81	0.97	198.40 [7]	/
**$Cl^- 2p_{1/2}$**	199.42	0.97	200.80 [8]	1.0
**Se LMM Auger**	198.34	6.5	/	/

S10. XPS Analysis of I 3d core levels

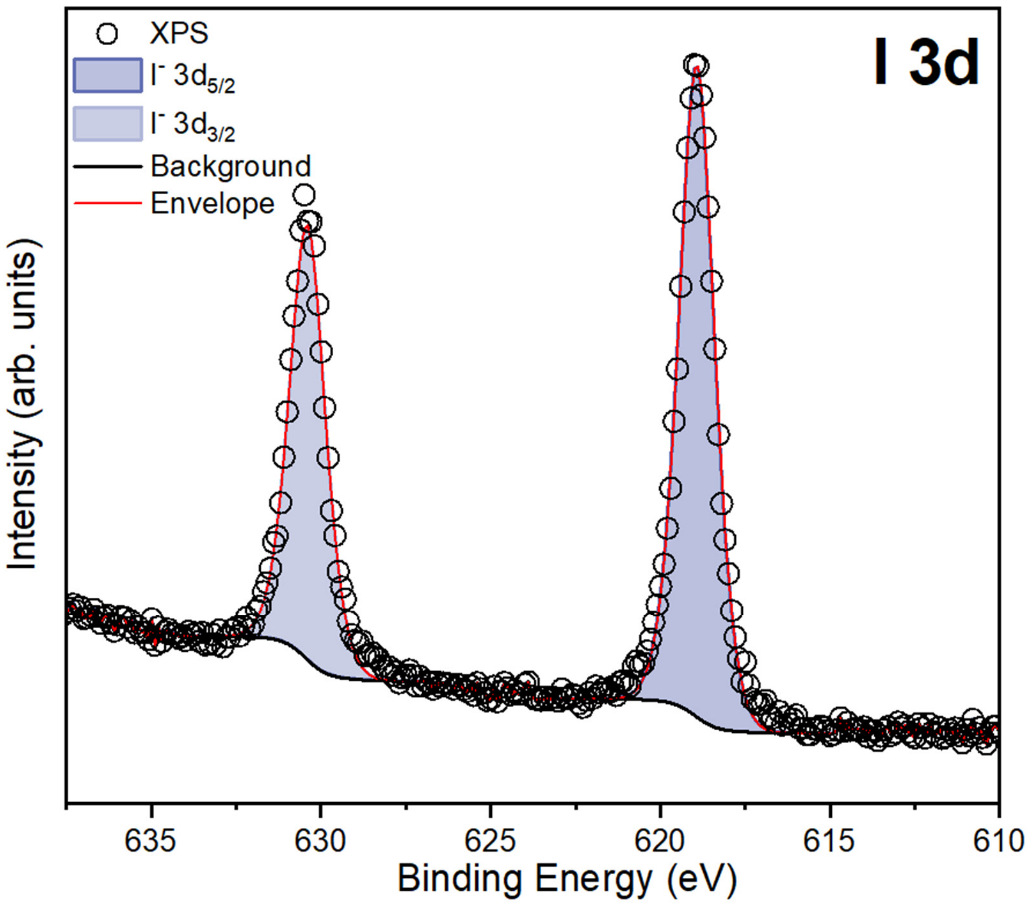

S11. Table of I 3d Binding Energies / Fitting Parameters

I 3d	Experimental		Literature	
Orbital	**Binding Energy (eV)**	**FWHM (eV)**	**Binding Energy (eV)**	**FWHM (eV)**
**I^- 3d$_{5/2}$**	618.93	1.23	/	/
**I^- 3d$_{3/2}$**	630.40	1.23	/	/

S12. Comparison of the unit cells of (i) $Cu_{7.4}Bi_6Se_{12}Cl_7$ [9] and (ii) $Cu_{7.62}Bi_6Se_{12}Cl_6I$, viewed along the *c* direction.

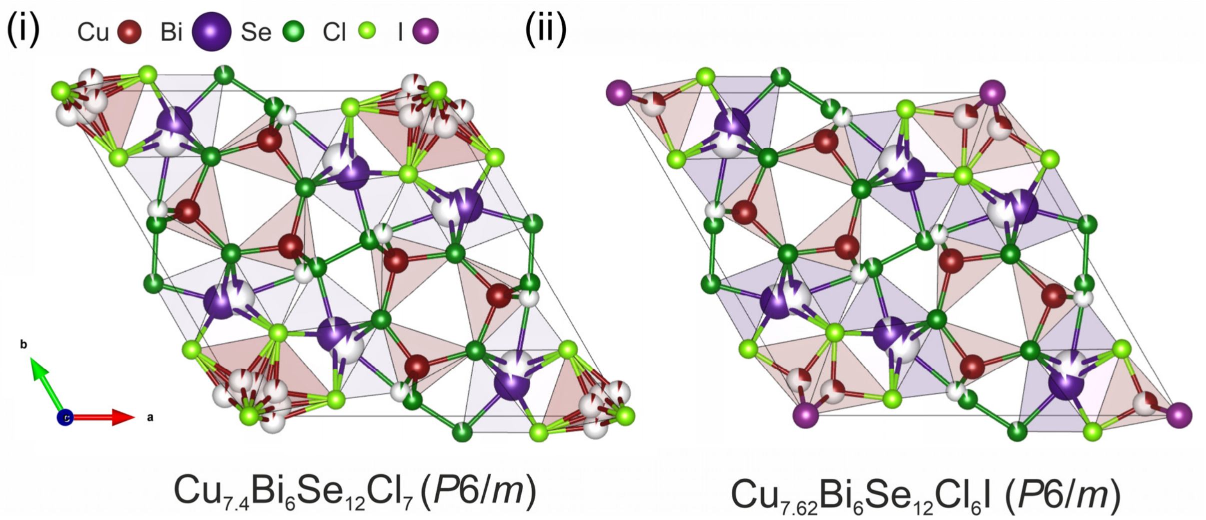

III. Powder X-Ray Diffraction

S13. Rietveld analysis of powder X-ray diffraction data collected on a capillary sample of $Cu_{7.62}Bi_6Se_{12}Cl_6I$ (Cu Kα_1 radiation (λ = 1.54056 Å)).

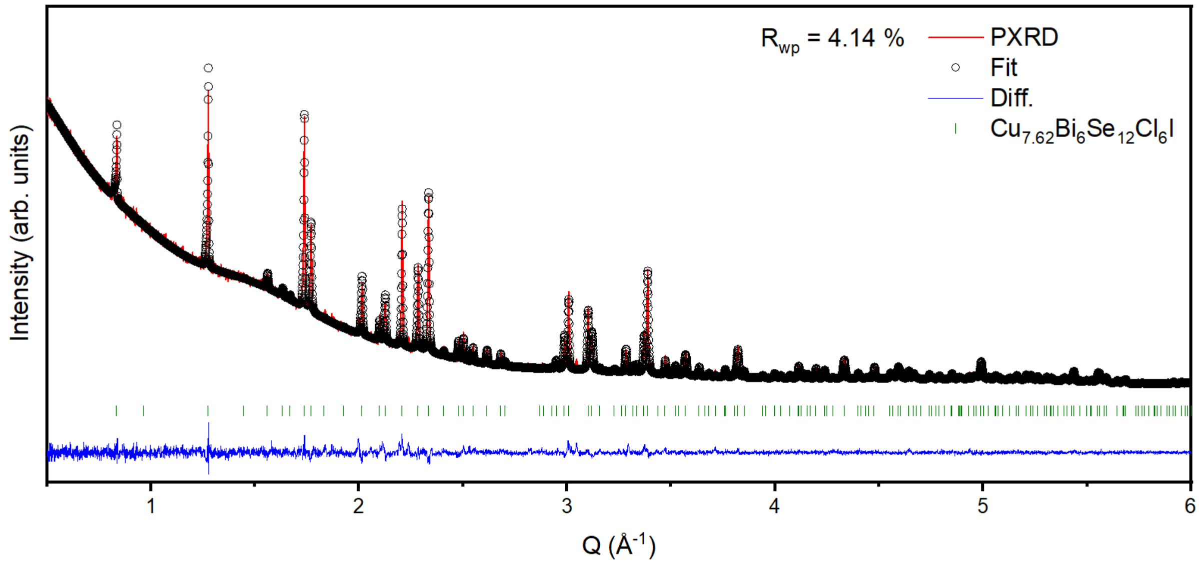

S14. Parameters from structure refinement against powder X-ray diffraction data measured on $Cu_{7.62}Bi_6Se_{12}Cl_6I$ at room temperature.

**Empirical Formula**	**$Cu_{7.62}Bi_6Se_{12}Cl_6I$**
Crystal System	Hexagonal
Space Group	*P*6/*m* (175)
Cell Parameters	$a = b$ = 15.073(2) Å<br>c = 4.0302(8) Å<br>α, β = 90° and γ = 120°
Cell Volume	792.939 Å^3
Density	6.181 g cm^{-3}
Pawley R_{wp}	3.76%
Rietveld R_{wp}	4.14%
R_{exp}	3.25%
R_p	2.75%
gof	1.27

Compositional Analysis

S15. Transmission Electron Microscopy (TEM) Images of $Cu_{7.62}Bi_6Se_{12}Cl_6I$ Particles

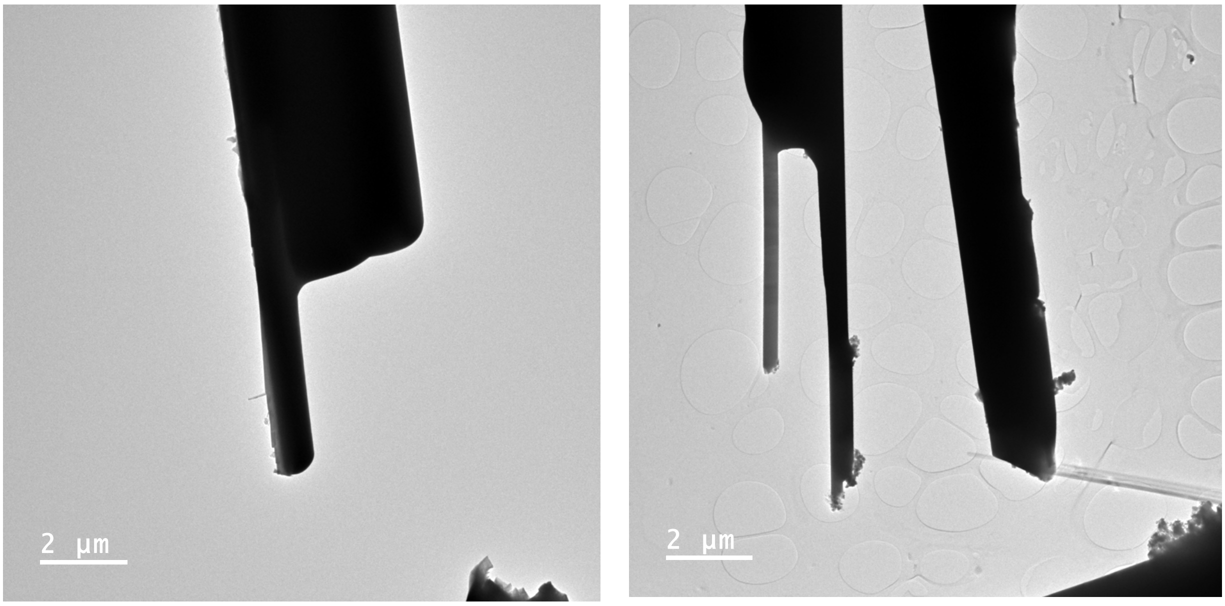

S16. SEM-EDX Imaging of the surface of a dense pellet of $Cu_{7.62}Bi_6Se_{12}Cl_6I$

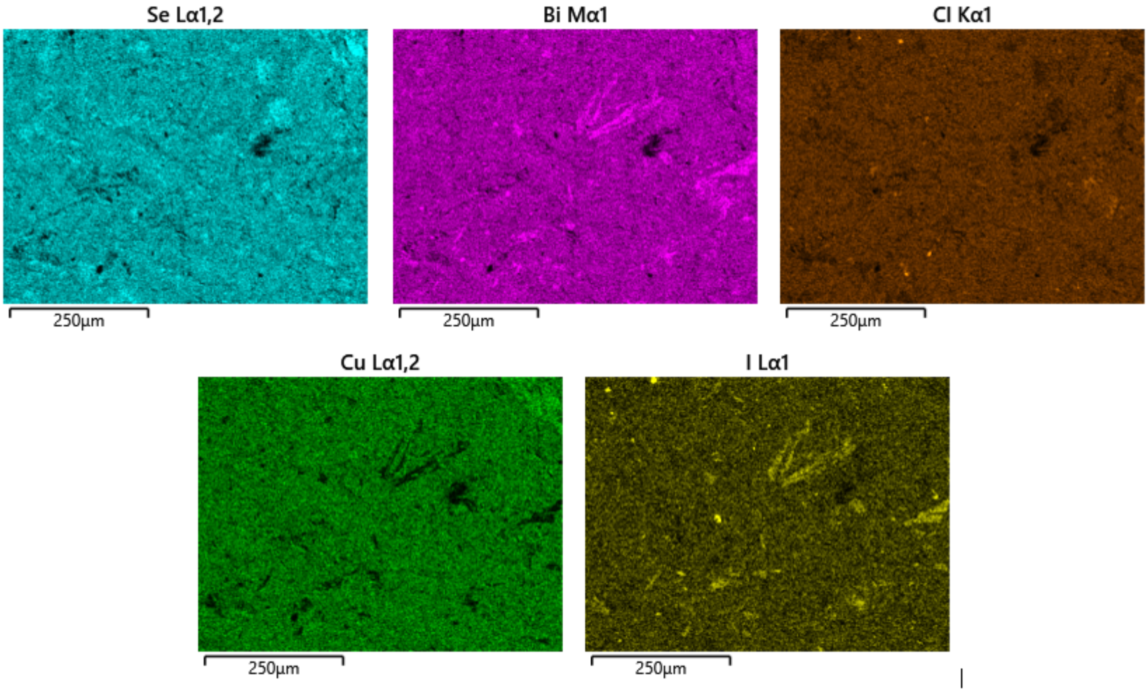

S17. SEM-EDX data analysis of Cu, Bi, Se and I measured on a cold-pressed pellet sample of $Cu_{7.62}Bi_6Se_{12}Cl_6I$.

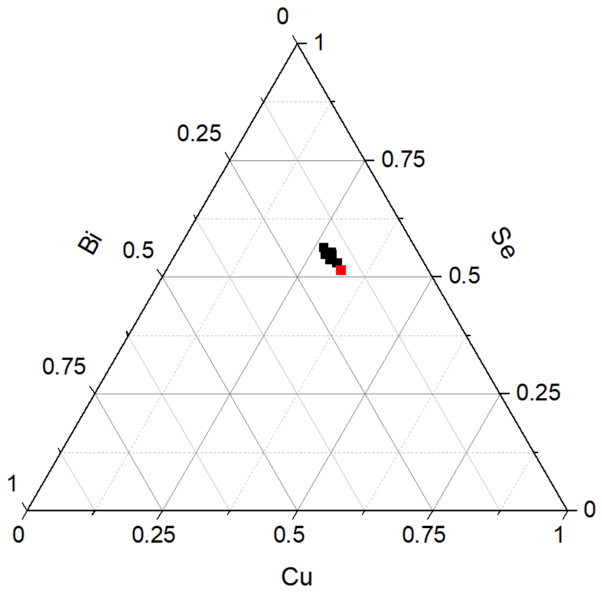

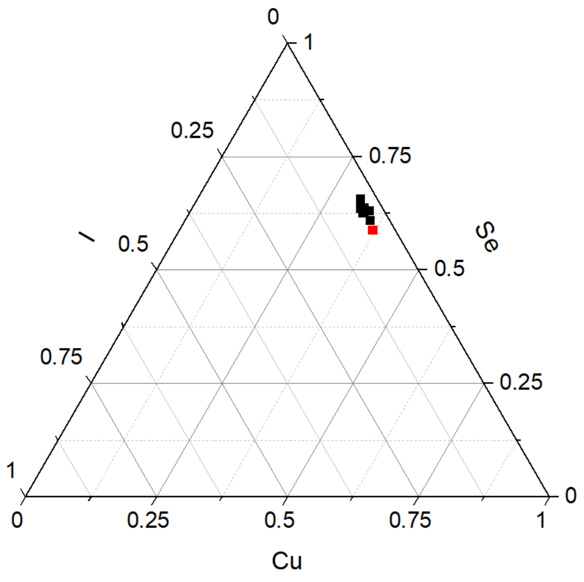

Environmental Stability

S18. PXRD data measured on $Cu_{7.62}Bi_6Se_{12}Cl_6I$ following exposure to ambient atmosphere at defined time intervals. The asterisk denotes the peak arising from a small contribution from the sample holder.

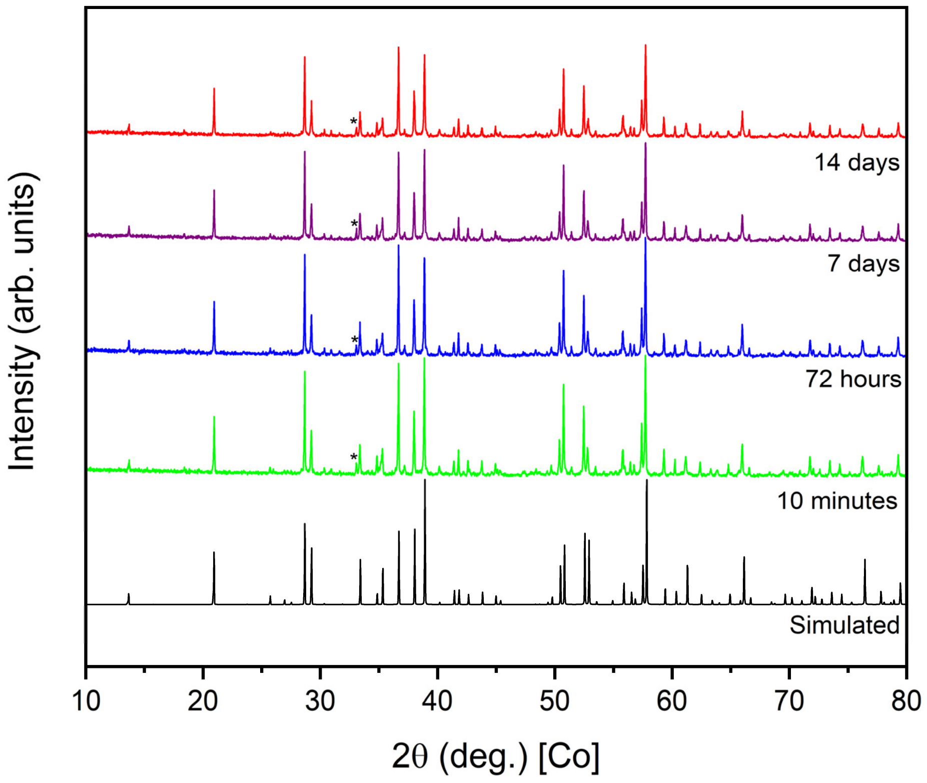

Electronic Structure

S19. Indirect band gap of $Cu_{7.62}Bi_6Se_{12}Cl_6I$ determined from Tauc analysis of Kubelka-Munk converted data.

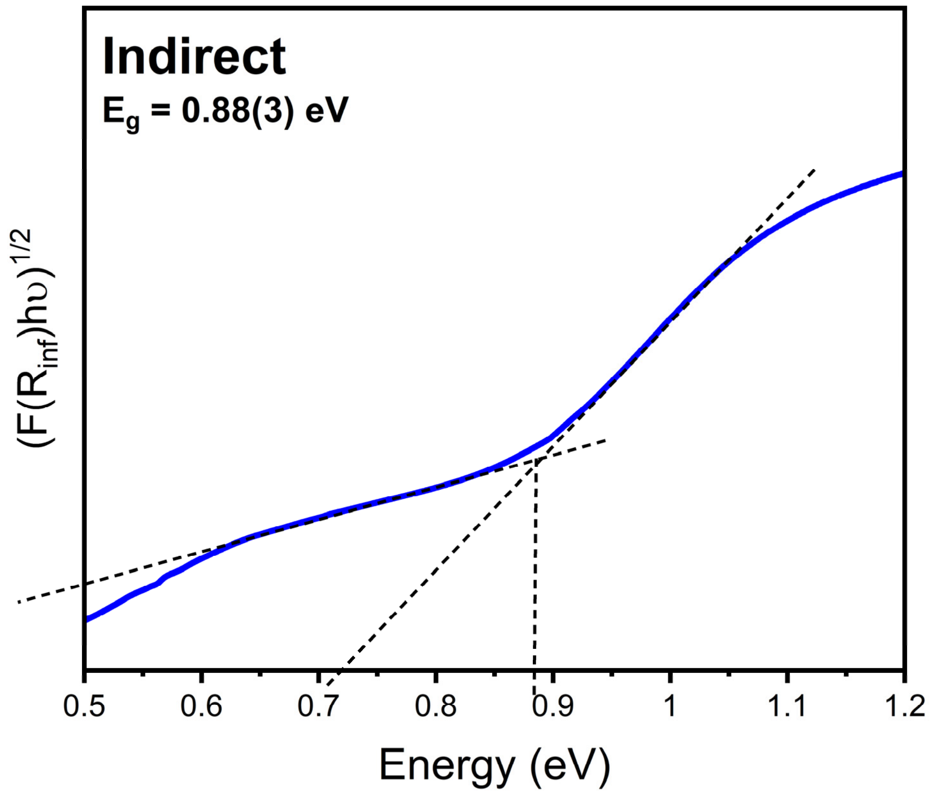

S20. Description of Band Alignment Determination

Band alignments for $Cu_{7.62}Bi_6Se_{12}Cl_6I$ were determined from measurement of the Secondary Electron Cut-off (SEC) region, shown in S21. The spectrometer work function (ϕ_s) was first determined from the SEC using Eq. 1.

$$1. \quad \phi_s = h\upsilon - E_{SEC}$$

Here, $h\upsilon$ is the photon energy and E_{SEC} is the energy of the onset of the SEC region. From this, the Ionisation Potential (IP) was calculated using Eq. 2.

$$2. \quad IP = \phi_s + E_{VBM}$$

Where E_{VBM} is the binding energy of the Valence Band Maximum (VBM). The band alignments can thus be calculated using the IP, shown in S22.

S21. Secondary Electron Cut-off (SEC) Region of $Cu_{7.62}Bi_6Se_{12}Cl_6I$

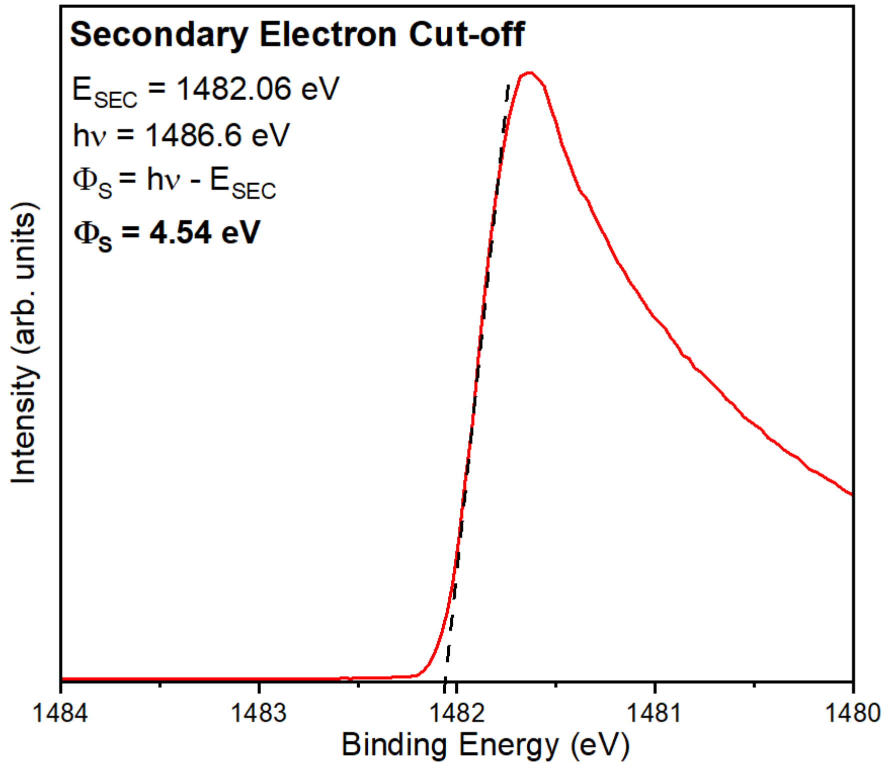

S22. Determination of Ionisation Potential using position of Valence Band Maximum (VBM)

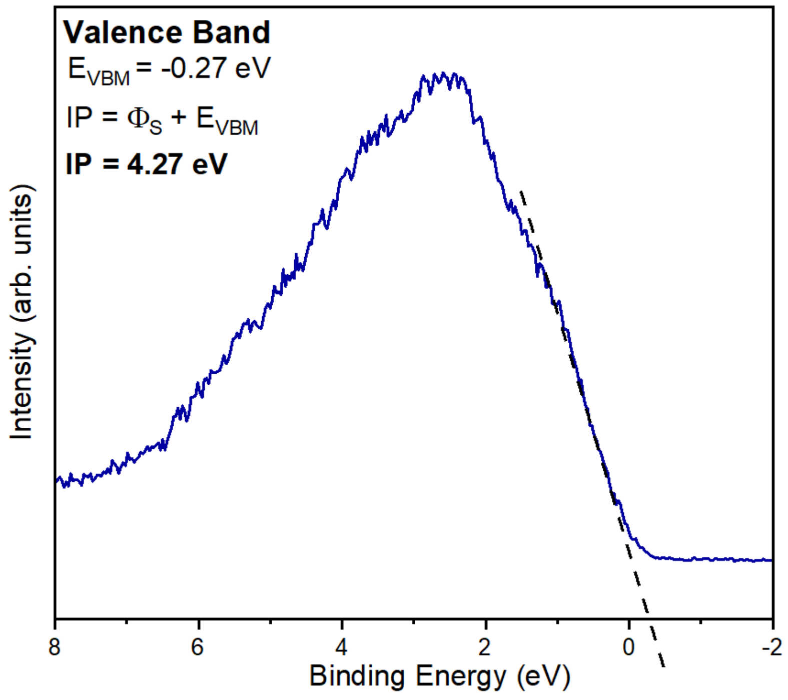

Thermal Properties

S23. Modelling of Specific Heat Capacity Data measured from $Cu_{7.62}Bi_6Se_{12}Cl_6I$ using (i) 1 Debye term (ii) 1 Debye and 1 Einstein term (iii) 2 Debye terms and 1 Einstein term. No linear contributions were used in the fitting shown here, and all Debye and Einstein prefactors summed to 1 in each case.

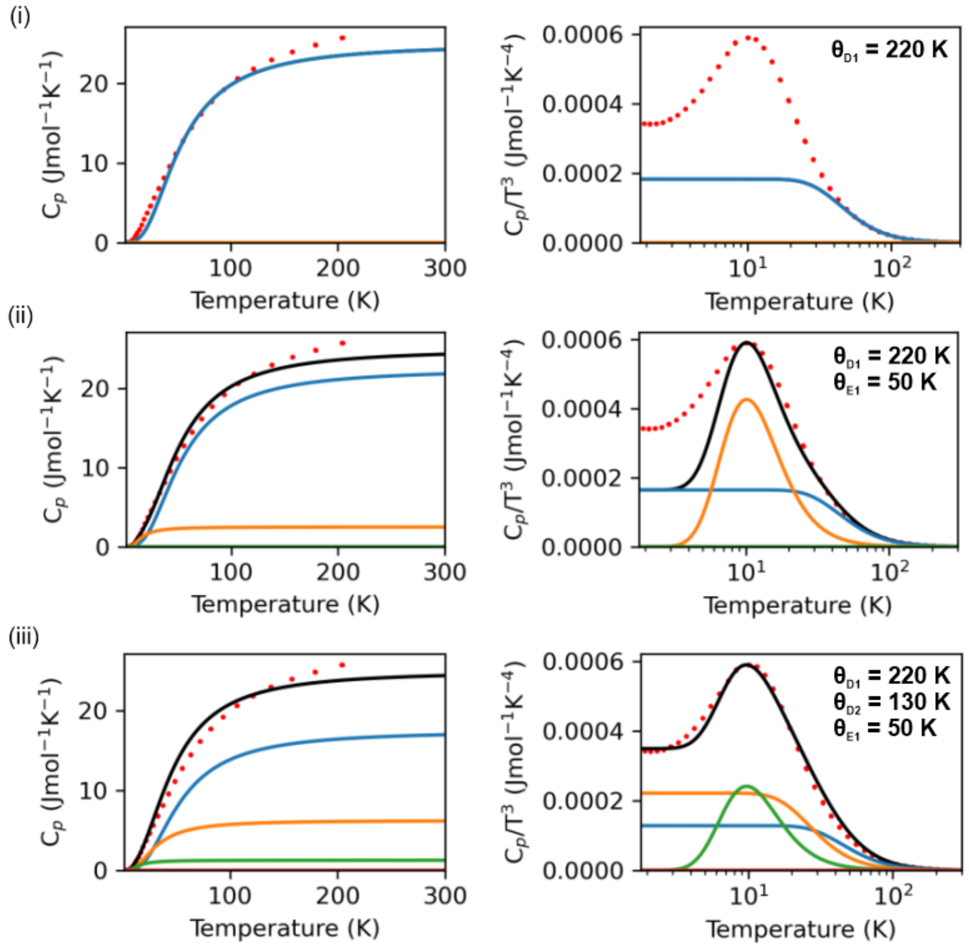

S23. Description of mathematical equations used for modelling of thermal conductivity data collected from a dense pellet of $Cu_{7.62}Bi_6Se_{12}Cl_6I$.

The minimum thermal conductivity (κ_{min}) of $Cu_{7.62}Bi_6Se_{12}Cl_6I$ was estimated using the Cahill model,[10] whereby the transfer of energy is enabled by the nearest neighbour interactions involved in a random walk of coupled quantum harmonic oscillators. This is described using three Debye integrals, which are taken over three sound modes (two transverse and one longitudinal) in Eq. 3.

$$3.\quad \kappa_{min} = \left(\frac{\pi}{6}\right)^{1/3} k_B n^{2/3} \sum_i v_i \left(\frac{T}{\Theta_i}\right)^2 \int_0^{\Theta_i/T} \frac{x^3 e^x}{(e^x-1)^2} dx$$

Where n is the number density of atoms, v_i is the speed of sound in each sound mode, and Θ_i is the cut-off frequency (Debye temperature) for each polarization:

$$\Theta_i = v_i \left(\hbar/k_B\right)(6\pi^2 n)^{1/3}$$

The diffuson-mediated thermal conductivity (κ_{diff}) was also calculated for $Cu_{7.62}Bi_6Se_{12}Cl_6I$ using the model developed by Agne et al.[11] Equation 4 approximates the limit of diffusive thermal conductivity in a material.

$$4.\quad \kappa_{diff}(T) \approx \frac{n^{-2/3} k_B}{2\pi^3 v_s^3} \left(\frac{k_B T}{\hbar}\right)^4 \int_0^{0.95\frac{\theta_D}{T}} \frac{x^5 e^x}{(e^x-1)^2} dx$$

Where v_s is the arithmetic average speed of sound and θ_D is the Debye temperature.

S24. Prefactors and Debye and Einstein frequencies used in the modelling of specific heat capacity data collected from $Cu_{7.62}Bi_6Se_{12}Cl_6I$.

**Compound**	θ_{D1} **(K)**	θ_{D2} **(K)**	θ_{E1} **(K)**	θ_{E2} **(K)**	**γ (Jmol^{-1}K^{-2})**
$\mathbf{Cu_{7.62}Bi_6Se_{12}Cl_6I}$	285(2)	130(1)	55(1)	28(1)	0.0001(1)
Prefactors	0.630	0.296	0.07	0.004	/

As required, the Debye and Einstein modelling prefactors sum to 1.

References

(1) Ismail, F. M.; Hanafi, Z. M. Some Physico-Chemical Properties of Bismuth Chalcogenides x-Ray Photoelectron and Diffuse Reflectance Spectra. *Z. Phys. Chem.* **1986**, *267O* (1), 667–672. DOI: 10.1515/zpch-1986-26782.

(2) Mytilineou, E.; Kounavis, P.; Chao, B. S. A study of n-type conduction in amorphous chalcogenide sputtered films. *J. Phys.: Condens. Matter* **1989**, *1* (28), 4687–4695. DOI: 10.1088/0953-8984/1/28/018.

(3) Rufus, I. B.; Ramakrishnan, V.; Viswanathan, B.; Kuriacose, J. C. Surface characterization of $CdS_{0.62}Se_{0.38}$ by X-ray photoelectron spectroscopy. *J. Mater. Sci. Lett.* **1992**, *11* (5), 252–254. DOI: 10.1007/bf00729403.

(4) Nelson, A. J.; Frigo, S. P.; Rosenberg, R. Valency and type conversion in $CuInSe_2$ with H_2 plasma exposure: A photoemission investigation. *J. Appl. Phys.* **1993**, *73* (12), 8561–8564. DOI: 10.1063/1.354063.

(5) Wright, M. A.; Lim, J.; Pacheco Muino, R. A.; Krowitz, A. E.; Hawkins, C. J.; Bahri, M.; Daniels, L. M.; Chen, R.; Gomes Chagas, L.; Cookson, J.; et al. Fast Mg-ion insertion kinetics in V_2Se_9. *J. Mat. Chem. A* **2024**, *12* (46), 32349–32358. DOI: 10.1039/d4ta04469j.

(6) Sobol, P. E.; Nelson, A. J.; Schwerdtfeger, C. R.; Stickle, W. F.; Moulder, J. F. Single Crystal $CuInSe_2$ Analysis by High Resolution XPS. *Surf. Sci. Spectra* **1992**, *1* (4), 393–397. DOI: 10.1116/1.1247638.

(7) Sesselmann, W.; Chuang, T. J. The interaction of chlorine with copper. *Surf. Sci.* **1986**, *176* (1-2), 32–66. DOI: 10.1016/0039-6028(86)90163-9.

(8) Vasquez, R. P. CuCl by XPS. *Surf. Sci. Spectra* **1993**, *2* (2), 138–143. DOI: 10.1116/1.1247732.

(9) Heerwig, A.; Ruck, M. $Cu_9Bi_9S_6Cl_8$ und $Cu_{7.4}Bi_6Se_{12}Cl_7$ - polyedernetzwerke mit dichalkogenidbrücken und mobilen kupfer(I)-kationen. *Z. Anorg. Allg. Chem.* **2009**, *635*, 2162–2169. DOI: 10.1002/zaac.200900361.

(10) Cahill, D. G.; Watson, S. K.; Pohl, R. O. Lower limit to the thermal conductivity of disordered crystals. *Phys. Rev. B.* **1992**, *46* (10), 6131–6140. DOI: 10.1103/physrevb.46.6131.

(11) Agne, M. T.; Hanus, R.; Snyder, G. J. Minimum thermal conductivity in the context of *diffuson*-mediated thermal transport. *Energy Environ. Sci.* **2018**, *11*, 609–616. DOI: 10.1039/c7ee03256k.
